# Immunotherapeutic IL-6R and targeting the MCT-1/IL-6/CXCL7/PD-L1 circuit prevent relapse and metastasis of triple-negative breast cancer

**DOI:** 10.7150/thno.92922

**Published:** 2024-03-03

**Authors:** Aushia Tanzih Al Haq, Pao-Pao Yang, Christopher Jin, Jou-Ho Shih, Li-Mei Chen, Hong-Yu Tseng, Yen-An Chen, Yueh-Shan Weng, Lu-Hai Wang, Michael P. Snyder, Hsin-Ling Hsu

**Affiliations:** 1 Institute of Molecular and Genomic Medicine, National Health Research Institutes, Miaoli, Taiwan.; 2 Department of Genetics, Stanford University School of Medicine, Stanford, CA, USA.; 3 Institute of Integrated Medicine and Chinese Medicine Research Center, China Medical University, Taichung, Taiwan.

**Keywords:** MCT-1, IL-6/IL-6R, CXCL7/CXCR2, PD-L1, Immunotherapy

## Abstract

**Rationale:** Multiple copies in T-cell malignancy 1 (MCT-1) is a prognostic biomarker for aggressive breast cancers. Overexpressed MCT-1 stimulates the IL-6/IL-6R/gp130/STAT3 axis, which promotes epithelial-to-mesenchymal transition and cancer stemness. Because cancer stemness largely contributes to the tumor metastasis and recurrence, we aimed to identify whether the blockade of MCT-1 and IL-6R can render these effects and to understand the underlying mechanisms that govern the process.

**Methods:** We assessed primary tumor invasion, postsurgical local recurrence and distant metastasis in orthotopic syngeneic mice given the indicated immunotherapy and MCT-1 silencing (shMCT-1).

**Results:** We found that shMCT-1 suppresses the transcriptomes of the inflammatory response and metastatic signaling in TNBC cells and inhibits tumor recurrence, metastasis and mortality in xenograft mice. IL-6R immunotherapy and shMCT-1 combined further decreased intratumoral M2 macrophages and T regulatory cells (Tregs) and avoided postsurgical TNBC expansion. shMCT-1 also enhances IL-6R-based immunotherapy effectively in preventing postsurgical TNBC metastasis, recurrence and mortality. Anti-IL-6R improved helper T, cytotoxic T and natural killer (NK) cells in the lymphatic system and decreased Tregs in the recurrent and metastatic tumors. Combined IL-6R and PD-L1 immunotherapies abridged TNBC cell stemness and M2 macrophage activity to a greater extent than monotherapy. Sequential immunotherapy of PD-L1 and IL-6R demonstrated the best survival outcome and lowest postoperative recurrence and metastasis compared with synchronized therapy, particularly in the shMCT-1 context. Multiple positive feedforward loops of the MCT-1/IL-6/IL-6R/CXCL7/PD-L1 axis were identified in TNBC cells, which boosted metastatic niches and immunosuppressive microenvironments. Clinically, *MCT-1^high^/PD-L1^high^/CXCL7^high^ and CXCL7^high^/IL-6^high^/IL-6R^high^ expression patterns predict worse prognosis and poorer survival of breast cancer patients.*

**Conclusion:** Systemic targeting the MCT-1/IL-6/IL-6R/CXCL7/PD-L1 interconnections enhances immune surveillance that inhibits the aggressiveness of TNBC.

## Introduction

Epithelial-to-mesenchymal transition (EMT) drives cancer metastasis by adapting to the microenvironment 1, floating through the blood or lymphatic circulation and settling in target organs 2. Enriched oncogenic factors enhance the capacities of self-renewing, pluripotent cancer stem cells (CSCs) 3, which drive tumorigenicity and therapeutic resistance. The cooperativity between innate and adaptive immunity establishes anticancer immunosurveillance 4, 5. Tumor-associated macrophages (TAMs) promote neoplasia, angiogenesis, inflammation and tissue remodeling 6, 7. Peripheral blood monocytes infiltrate the stroma and differentiate into either M1 (tumoricidal) or M2 (protumor) macrophages upon stimulation 8, 9. Lipopolysaccharide (LPS) or Th1 cytokines such as interferon (IFN)-γ and granulocyte-macrophage colony-stimulating factor activate M1 macrophages, which produce proinflammatory cytokines, such as interleukin (IL)-1β, IL-6, IL-12, IL-23 and tumor necrosis factor-α. Th2 cytokines, such as IL-4, IL-10 and IL-13, activate M2 macrophages that secrete the anti-inflammatory cytokines IL-10 and transforming growth factor beta (TGF-β). M2 macrophages reduce tumor-infiltrating lymphocytes (TILs) and induce Tregs, which compromise CD4(+) helper and CD8(+) cytotoxic T (CTLs), NK and antigen-presenting cells 10.

TNBCs lose estrogen receptor (ER), progesterone receptor (PR) and human epidermal growth factor receptor 2 (HER2), showing a robust immunosuppressive tumor microenvironment (TME) and high invasiveness and mortality 11, 12. Programmed cell death protein 1 (PD-1) on CTLs interacts with PD-1 ligand (PD-L1) (also known as B7-H1 antigen or CD274) on tumor cells, then CTLs exhaust cytotoxicity to fight against tumors 13. PD-L1 stimulates T cells to produce IL-10, which activates M2 macrophages 14, but PD-L1-based immunotherapy repolarizes TAMs toward the M1 phenotype 15. In addition, crosstalk between TNBC cells and monocytes induces chemokine (C-X-C motif) ligand 7 (CXCL7) secretion from monocytes to enrich M2 macrophages 16, indicating that chemokines such as CXCL7 regulate the immune landscape.

PD-1/PD-L1-based immunotherapies alone or in combination with other systemic treatments improve TNBC therapy 17. Combined IL-6 and PD-L1 immunotherapies has been reported to reduce pancreatic tumors and hepatoma in mice 18, 19. Anti-IL-6 enhances Th1 immune responses and decreases PD-L1 levels and melanoma development in mice 20. While anti-PD-L1 alone cannot enhance the Th1 response, combined immunotherapies against IL-6 and PD-1/PD-L1 induce T-cell-attracting chemokines and promote IFNγ-producing CD4(+) T-cell infiltration, synergistically enhancing therapeutic efficacy.

MCT-1 (also known as MCTS1) regulates translation reinitiating and ribosomal recycling 21-24. In complex with density-regulated protein and transfer RNA 21, 25, 26, MCT-1 binds the 40S ribosome to initiate noncanonical translation in organogenesis 23 and oncogenesis 24. We found that MCT-1 relocates at the mitotic centrosome and midbody 27. Induced MCT-1 accelerates destabilization of p53 and PTEN 28-30, damages cytokinesis and chromosomes 28, 30 and provokes *reactive oxygen species* 31. But MCT-1 depletion prevents catastrophic mitosis, genomic aberrations and cancer growth 27, 29, 30, 32. Previously, we reported that MCT-1 protein was enriched in 70.8% of TNBC patients, and overexpressing MCT-1 promoted IL-6/IL-6R/gp130 signaling-mediated EMT and TNBC progression in xenograft mice 33. MCT-1 and IL-6 synergistically promote the plasticity of breast CSCs (BCSCs) and M2 macrophages; thus, targeting MCT-1 advances the effect of an IL-6 receptor (IL-6R) monoclonal antibody (mAb) (tocilizumab) against BCSCs and M2 polarization. Tocilizumab binds both soluble and membrane-bound IL-6R, which prevents IL-6/IL-6R signaling function 34. Dual therapies involving tocilizumab are superior to monotherapy. For instance, tocilizumab alongside maraviroc (a CCR5 inhibitor) blunts thoracic metastasis in TNBC xenograft mice 35, potentiates cytotoxicity of cisplatin and suppresses BCSCs and tumorigenesis in humanized orthotopic TNBC mice 36.

Here, we first identify positive feedforward interconnections of the MCT-1/IL-6/IL-6R/CXCL7/PD-L1 nexus in TNBC cells. Primary TNBC progression and postsurgical recurrence/metastasis in xenograft mice were efficiently inhibited by MCT-1 knockdown (shMCT-1) and IL-6R immunotherapy. Surprisingly, sequential immunotherapy of PD-L1 and IL-6R successfully reduced TNBC aggressiveness and mortality, particularly in the shMCT-1 milieu. Our results establish an innovative and systemic therapy for TNBC that may enhance clinical impacts and patient survival.

## Results

### Loss of MCT-1 prevents TNBC growth and the tumor microenvironment

**Figure 1:**
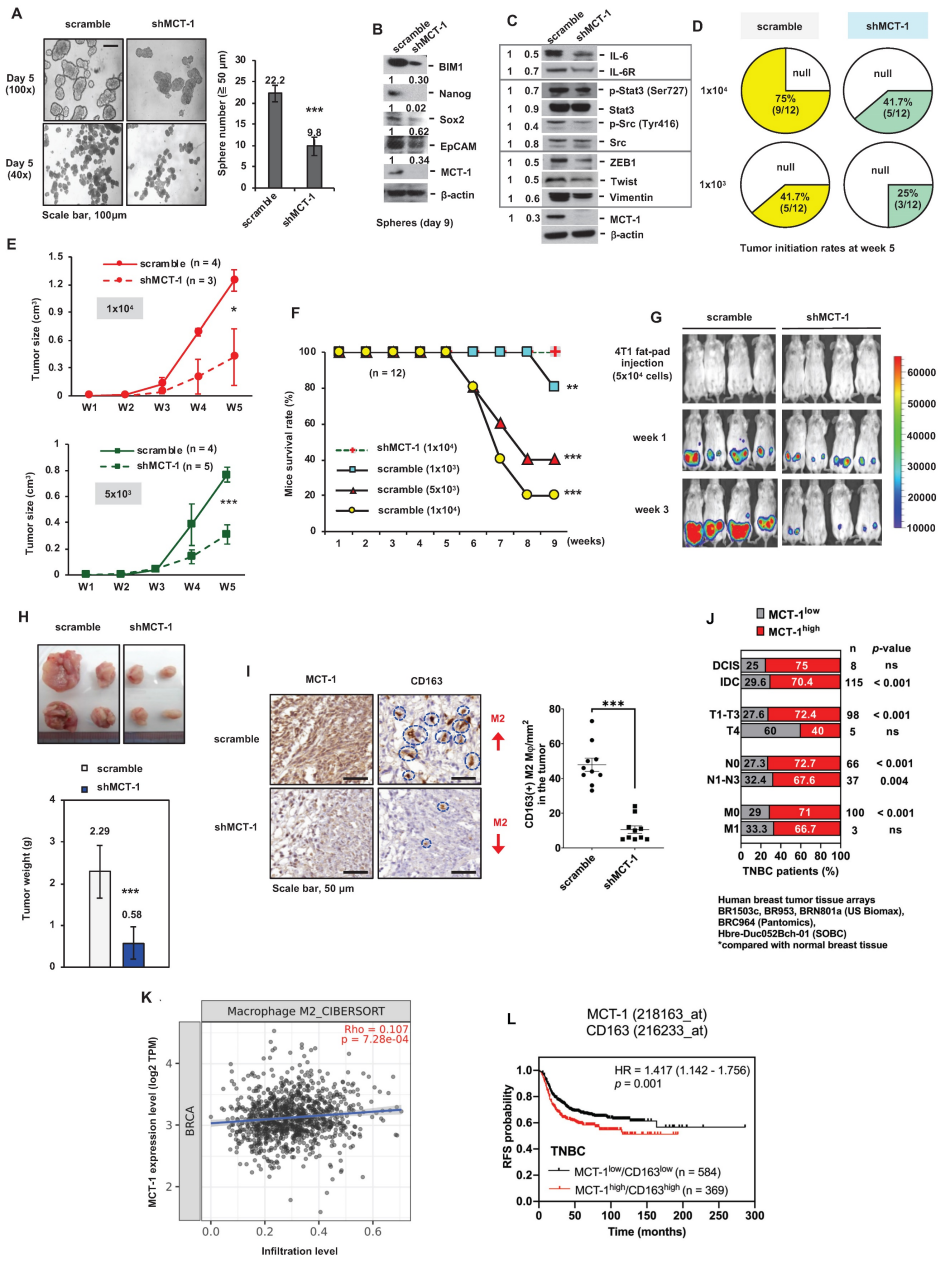
** MCT-1 knockdown prevents TNBC growth and M2 macrophage infusion.** TNBC 4T1 cell mammosphere formation (Day 5) (≥ 50 μm) was compared between the unsilenced (scramble) and the MCT-1 silencing (shMCT-1) groups (n = 25) (A). Scale bars, 100 μm. Stemness markers in day-9 mammospheres (B) and the indicated protein levels (C) were detected. Mammary tumor incidences were monitored over 5 weeks after orthotopically implanting 4T1 cells (1x10^3^, 1x10^4^) into BALB/c mice (n = 12). Nontumorigenic (null) (D). After injecting 4T1 cells (1x10^4^, 5x10^3^) into mice, breast tumor volume changes for 5 weeks (n = 3 ~ 5) (E) and survival rates for 9 weeks (n = 12) (F) were checked. IVIS detected mammary tumorigenesis (G) and tumor weights (H) were measured in 4T1 cell (5x10^4^)-bearing mice (n = 4) in week 4. Intratumoral CD163(+) M2 macrophages (indicated by circles) were measured in randomly selected fields (n = 10) for each group (n = 3) (I). Scale bars, 50 μm. Proportion (%) of TNBC patients with low (MCT-1^low^) and high MCT-1 (MCT-1^high^) protein was stratified by breast cancer types (ductal carcinoma in situ (DCIS) and invasive ductal carcinoma (IDC)), tumor size (T1-T4), lymphatic (N0-N3) and distant metastasis stages (M0-M1) (J). Significance was defined by comparing the cohorts with normal breast tissue assembled in BR1503c, BR953, BRN801a (US Biomax), BRC964 (Pantomics) and Hbre-Duc052Bch-01 (SOBC) human tissue arrays. TIMER 2.0 and the CIBERSORT-ABS algorithm were used to evaluate the correlation of MCTS1 (MCT-1) expression in the TCGA-BRCA cohort with the infiltration of M2 macrophages. Log2 transcripts per million (TPM) indicate expression levels (K). The Kaplan‒Meier plotter breast cancer database was used to analyze RFS associated with MCT-1/CD163 expression patterns in TNBC patients (L). All values are indicated by mean values. Error bars reflect the standard error of the mean (SEM). Statistical significance was determined by a two-tailed unpaired Student's *t* test (A, E, H, I), two-sided Fisher's exact test (J), Spearman's (⍴) correlation analysis (K) and multivariate Cox regression analysis coupled with log-rank test (L). ns: not significant, *p < 0.05, **p < 0.01, ***p < 0.001.

The TNBC 4T1 cell line derived from a spontaneous BALB/c mammary tumor is negative for ER, PR and HER2 37. To study loss-of-function of MCT-1, short hairpin RNA (shRNA) was used to silence the MCT-1 gene (shMCT-1) in 4T1 cells and greatly reduced the high cancer stemness features of *mammosphere* formation (Figure 1A) and expression levels of stem cell markers (BIM-1, Nanog, Sox-2, EpCAM) (Figure 1B), EMT inducers (ZEB1, Twist, vimentin**)** and oncogenic factors (IL-6/IL-6R, p-Stat3 (Ser727), p-Src (Tyr416)) (Figure 1C) compared with the unsilenced cells (scramble).

CSCs are self-renewing, heterogenic and tumorigenic subpopulations 38. To examine the effect of putative *breast CSCs (BCSCs)*, different amounts of 4T1 cells (1x10^4^ and 1x10^3^) were orthotopically implanted into the mammary fat pads of immunocompetent BALB/c mice. During the 5-week injection, the tumorigenicity of shMCT-1 cells (41.7% and 25%) was correspondingly lower than that of *scramble cells (75% and 41.7%) when equal cell doses were compared* (Figure 1D). The tumor growth rates of shMCT-1 cells (5x10^3^ and 1x10^4^) were also much more decreased compared with those of xenografts established with equal amounts of scramble cells (Figure 1E). The mouse survival probability in week 9 was enhanced while implanting a higher dose of shMCT-1 cells (1x10^4^), but mice bearing lower doses of scramble cells (5x10^3^ and 1x10^4^) exhibited far shorter survival because tumor aggressiveness caused death and morbidity (Figure 1F). Furthermore, the mice were implanted with pGL3-luciferase reporter-engineered 4T1 cells (5x10^4^) and monitored by an *in vivo* imaging system (IVIS), demonstrating that tumor intensity was reduced dramatically in the shMCT-1 group (Figure 1G). Four weeks postinjection, the average tumor mass was much less in the shMCT-1 group (~ 0.58 g) compared with the scramble group (~ 2.29 g) (Figure 1H). Fewer CD163(+) M2 macrophages were detected in the shMCT-1 tumor than the scramble tumor (Figure 1I).

By comparing human TNBC cases with normal breast tissue 33, we further confirmed that high amount of MCT-1 protein significantly correlated with invasive ductal carcinoma (IDC) cancer type, T1-T3 tumor stage and lymph node metastasis (N1-N3) (Figure 1J). Alongside ductal carcinoma in situ (DCIS), minor association was found in TNBC cases with T4 and distant metastasis (M1) stages (Figure 1J). The Cancer Genome Atlas of Breast Invasive Carcinomas (TCGA-BRCA) analysis also indicated that high MCTS1 (MCT-1) expression levels were positively associated with M2 macrophage infiltration (Figure 1K). The Kaplan‒Meier plotter database further supports that the MCT-1^high^/CD163^high^ expression pattern represents decreased relapse-free survival (RFS) in TNBC patients (Figure 1L). Therefore, shMCT-1 treatment suppresses TNBC stemness, tumorigenicity, invasiveness, metastasis initiation, microenvironment and mortality.

### Abolishing MCT-1 and IL-6/IL-6R inhibit inflammation and proliferation pathways

**Figure 2:**
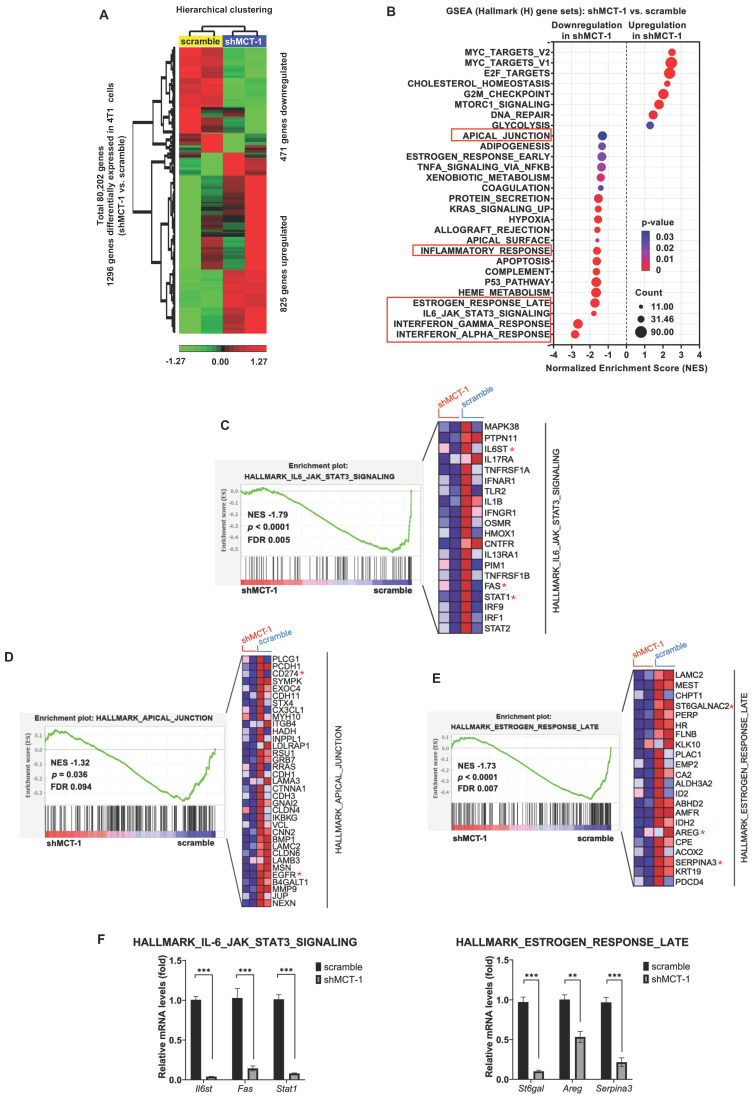
** The inflammatory-associated pathways are suppressed by shMCT-1 treatment.** (A) Hierarchical clustering of DEGs from two biologically independent samples of 4T1 cells (red, upregulated; green, downregulated). Data statistics were based on an ANOVA test with a fold change ≥ 1.2 or < -1.2 (*p* < 0.05). (B) The bubble plot showed GSEA using Hallmark (H) gene sets that elucidated all significantly altered pathways in shMCT-1 vs. scramble. Normalized enrichment score (NES). False discovery rate (FDR) < 25%. Bubble size is proportional to the number (count) of core genes within the pathway as identified by the leading-edge subsets. Color intensity represents statistical significance. (C) Enrichment plot (left) of the Hallmark IL-6/JAK/STAT3 signaling in shMCT-1 vs. scramble. The expression of all core genes suppressed by shMCT-1 in the IL-6/JAK/STAT3 signaling identified from the leading-edge gene list from GSEA was plotted in a heatmap (right) (red, upregulation; blue, downregulation; color intensity indicates the strength of regulation). (D-E) Enrichment plots (left) and heatmaps (right) of representative core genes within the Hallmark apical junction complex (D) and late response of estrogen (E) from GSEA. (F) The 4T1 tumors (n = 5) were used to validate representative core genes within the IL-6/JAK/STAT3 signaling (*Il6st*, *Fas* and *Stat1*) (marked with a red asterisk (*)) and late response of estrogen (*St6gal*, *Areg* and *Serpina3*). The relative mRNA levels were normalized to β-actin and then compared with that in the scramble cells. Statistical significance was determined by gene set-based permutation test (B-E) and two-tailed unpaired Student's *t* test (F). ***p < 0.001.

To identify the downstream genes that were deregulated after MCT-1 silencing, the transcriptome of 4T1 cells was studied using microarray analysis. Unsupervised hierarchical clustering with a statistical threshold of a fold change ≥ 1.2 (*p*-value < 0.05) identified 1,296 differentially expressed genes (DEGs) in the shMCT-1 cells compared with the scramble cells (Figure 2A, Table S1). Of these DEGs, shMCT-1 downregulated 471 genes (36.34%) and upregulated 825 genes (63.66%). Gene set enrichment analysis (GSEA) using Hallmark (H) gene sets indicated that the response of interferon-alpha (IFNα) and interferon-gamma (IFNγ), the IL-6/JAK/STAT3 pathway, and the late response of estrogen were the most underrepresented in the shMCT-1 context (Figure 2B, Table S2). Leading-edge gene list predicted a panel of 20 core genes in the IL-6/JAK/STAT3 pathway (24%) that were suppressed by shMCT-1 (NES = -1.79, p < 0.0001) (Figure 2C, Table S2). Within core genes in apical junction gene sets (28%) (Figure 2D, Table S2), we also observed de-enrichment of PD-L1 (CD274) and receptor tyrosine kinase EGFR in shMCT-1 (NES = -1.32, p = 0.036). This was consistent with prior reports in lung cancer demonstrating that EGFR is downstream of MCT-1 31. Silencing MCT-1 also hampered EGFR ligand, AREG 39, and glycosylation regulators of EGFR and PI3K/AKT 40, 41, sialyltransferase families ST6Gal/ST6GalNAc (Figure 2E, Table S1), as revealed by core genes in the late response of estrogen (30%) (NES = -1.73, p < 0.0001). We further validated that the representative core genes in the IL-6/JAK/STAT3 pathway (*Il6st* (*Gp130*), *Fas* and *Stat1*) and the late response of estrogen (*St6gal*, *Areg* and *Serpina3*) were highly reduced in shMCT-1 tumor when compared to scramble tumor (Figure 2F). Of note, shMCT-1 also suppressed core genes contributing to responses to IFNα (53%) (NES = -2.82, p < 0.0001) (Table S2, Figure S1A), IFNγ (30%) (NES = -2.66, p < 0.0001) (Table S2, Figure S1B) and inflammation (19%) (30%) (NES = -1.62, p < 0.0001) (Table S2, Figure S1C). Interferons have been reported to induce EGFR, PD-L1 and IL-6 42-45. Thus, depleting MCT-1 inhibits inflammation-associated and survival pathways in metastatic TNBC.

**Figure 3:**
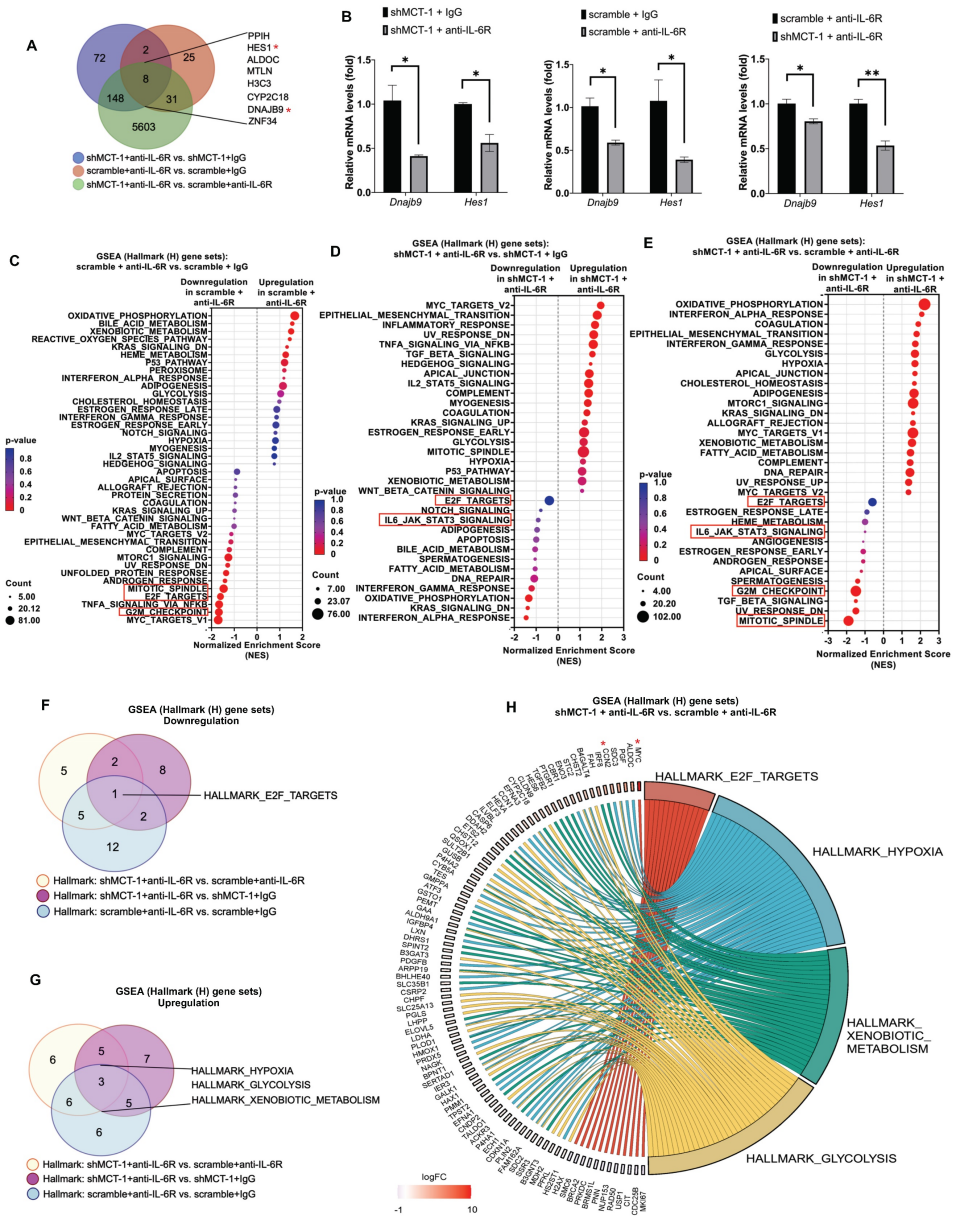
** shMCT-1 and anti-IL-6R immunotherapy mutually hinder proliferation pathways.** (A) Venn diagram represented unique DEGs among anti-mouse IL-6R Ab- (anti-IL-6R-) or IgG2b, κ-(IgG-) treated shMCT-1 (shMCT-1 + anti-IL-6R vs. shMCT-1 + IgG, blue), anti-IL-6R- vs. IgG-treated scramble (scramble + anti-IL-6R vs. scramble + IgG, red) and shMCT-1 vs. scramble within anti-IL-6R treatment (shMCT-1 + anti-IL-6R vs. scramble + anti-IL-6R, green). List of overlapped genes was shown. DEGs were identified by EdgeR and analyzed by Fisher's exact test with a *p-*value < 0.05. Genes marked with red asterisk denote representatives for validation experiment. (B) Quantitative RT-PCR analysis of representative common targets, *Dnajb9* and *Hes1*, in anti-IL-6R- or IgG-treated 4T1 shMCT-1 and scramble cells (n = 4). The relative mRNA levels were normalized to β-actin. Statistical significance was determined by a two-tailed unpaired Student's *t* test and corrected with the Holm-Sidak test. (C-E) The bubble plot showed GSEA using Hallmark (H) gene sets that elucidated all altered pathways in scramble + anti-IL-6R vs. scramble + IgG (C), shMCT-1 + anti-IL-6R vs. shMCT-1 + IgG (D) and shMCT-1 + anti-IL-6R vs. scramble + anti-IL-6R (E). (F-G) Venn diagrams showing common downregulated (F) and upregulated (G) pathways among shMCT-1 + anti-IL-6R vs. scramble + anti-IL-6R (light yellow), shMCT-1 + anti-IL-6R vs. shMCT-1 + IgG (magenta) and scramble + anti-IL-6R vs. scramble + IgG (light blue) analyzed by GSEA. Gene set name was shown. (H) Shared pathways (right) between shMCT-1 and anti-IL-6R alongside with their core genes with log2 fold change (Log2FC) ≥ 0.6 or < -0.6 (left) were depicted by chord plot. Fold change and gene membership were based on shMCT-1 + anti-IL-6R vs. scramble + anti-IL-6R (red, upregulation; blue, downregulation; color intensity indicates the strength of regulation). Right-to-left connections indicated associated core genes in the given pathway as shown by the leading-edge subsets. *p < 0.05, **p < 0.01.

Our previous work and Figure 2B identified that MCT-1 mediated IL-6/IL-6R/gp130 signaling 33. We thus treated 4T1 cells with anti-mouse IL-6R immunotherapy (clone 15A7), which blocks IL-6 binding to IL-6Rα 46, thus consequently inhibits both IL-6 classic and trans-signaling. By performing RNA sequencing analysis, nearly 10,599 transcripts were uncovered (Table S3A). We further did overlap analysis between 66 unique DEGs in scramble (scramble + anti-IL-6R vs. scramble + IgG) (Table S3B) and 230 unique DEGs in shMCT-1 (shMCT-1 + anti-IL-6R vs. shMCT-1 + IgG) (Table S3C) following anti-IL-6R in comparison to IgG as well as 5,790 unique DEGs in shMCT-1 vs. scramble within anti-IL-6R treatment (shMCT-1 + anti-IL-6R vs. scramble + anti-IL-6R) (Table S3D), resulting only 8 genes as common targets of both shMCT-1 and anti-IL-6R (Figure 3A), which were PPIH, HES1, ALDOC, MTLN, H3C3, CYP2C18, DNAJB9 and ZNF34. These common targets were majorly involved in biological regulation, cellular process and metabolic process (Figure S2B, Table S4). Of interest, as indicated by Venn diagram (Figure 3A) and unsupervised hierarchical clustering (Figure S2A), unique transcripts were predominantly contributed by shMCT-1 rather than anti-IL-6R immunotherapy. This was consistent with previous report demonstrating that IL-6 is downstream of MCT-1 33, thus targeting IL-6R consequently has similar effect to shMCT-1. Complemented with RNA sequencing results (Figure 3A), *Dnajb9* and *Hes1* were downregulated by both anti-IL-6R and shMCT-1 when validated by quantitative RT-PCR (Figure 3B).

GSEA with Hallmark (H) gene sets was then employed to gain insights into altered pathways between scramble and shMCT-1 upon anti-IL-6R compared to those treated by IgG (scramble + anti-IL-6R vs. scramble + IgG and shMCT-1 + anti-IL-6R vs. shMCT-1 + IgG) (Figure 3C-D). Anti-IL-6R treatment in scramble most significantly hindered genes involved in cell cycle such as during G2/M checkpoint (33%) (NES = - 1.68, p < 0.0001) and mitotic spindle assembly (37%) (NES = - 1.47, p = 0.002) (Figure 3C, Table S5A). Combined with shMCT-1, anti-IL-6R mostly suppressed inflammation-associated pathways (Figure 3D, Table S5B), marked with significant de-enrichment from IFNα response (23%) (NES = -1.46, p = 0.028) and minor reduction in IFNγ response (20%) (NES = -1.21, p = 0.145) and the IL-6/JAK/STAT3 signaling (20%) (NES = -0.91, p = 0.611). When shMCT-1 compared to scramble within anti-IL-6R treatment (shMCT-1 + anti-IL-6R vs. scramble + anti-IL-6R) (Figure 3E, Table S5C), we observed similar underrepresentation of genes involved in the IL-6/JAK/STAT3 signaling (10%) (NES = -1.04, p = 0.379), followed by larger reduction of genes acting in the mitotic spindle (39%) (NES = -1.88, p < 0.0001) and G2/M checkpoint (42%) (NES = -1.46, p = 0.003). Among triple comparison of these perturbed gene sets, overlap analysis identified transcription factor E2F targets, which regulating cell cycle progression, as the only common downregulated pathway by shMCT-1 and anti-IL6R (Figure 3F). This indicated that targeting MCT-1 and IL-6/IL-6R both exerted anti-proliferative effects, as evidenced separately by earlier investigations 27, 36. Additionally, three pathways were found to be mutually upregulated by shMCT-1 and anti-IL-6R (Figure 3G), including response to hypoxia and metabolic processes such as glycolysis and metabolism of xenobiotics. Accordingly, genes linked to the enrichment of hypoxia, glycolysis and metabolism of xenobiotics were upregulated in anti-IL-6R-treated shMCT-1 as compared to anti-IL-6R-treated scramble cells (Figure 3H), whereas most core genes associated with E2F targets were downregulated. Upregulation of MYC (Figure 3H), as component of E2F targets gene set, was likely a mechanism exploited by shMCT-1 and anti-IL-6R to sensitize TNBC cells to apoptosis. MYC has been shown to promote apoptosis during hypoxia and nutrient deprivation 47, as supported by increased expression of hypoxia and glycolysis genes (Figure 3H). Upregulation of MYC could be corroborated by quantitative RT-PCR analysis in both human and mouse TNBC cell lines with shMCT-1 and anti-IL-6R treatment (Figure S2C), but upregulation of CCN2, one of core genes in hypoxia, could not be observed in human TNBC cells, perhaps because human and mouse CCN2 did not share equivalent functional properties. Collectively, these results suggest that shMCT-1 treatment blocks IL-6 signaling transduction, cell proliferation and oncogenesis.

### shMCT-1 prevents primary TNBC progression and advances IL-6R immunotherapy against recurrence and metastasis after surgery

**Figure 4:**
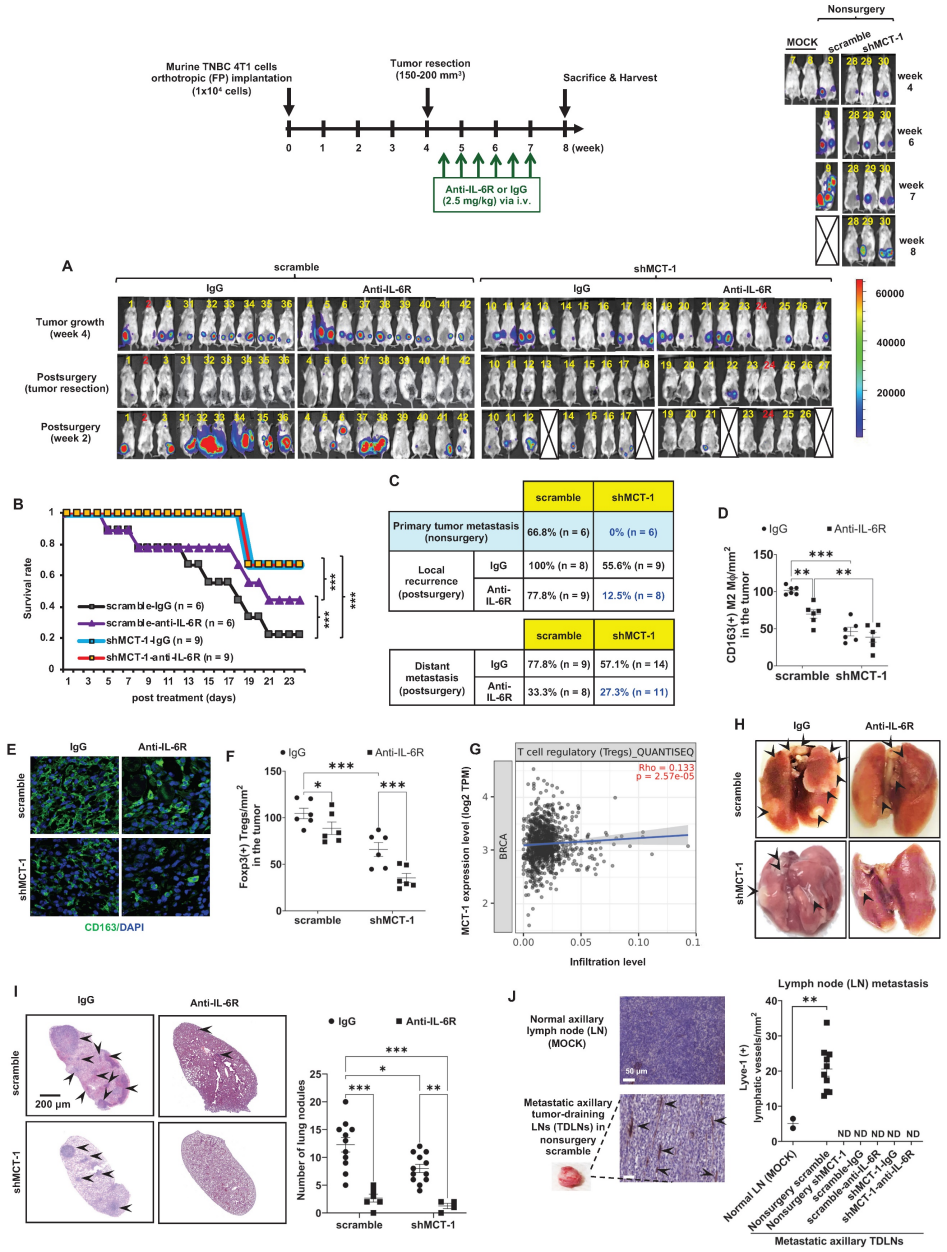
** shMCT-1 enhances IL-6R-based immunotherapy against TNBC recurrence and metastasis.** (A) The 4T1 cells (1x10^4^) were orthotopically implanted into BALB/c mice. The resulting breast tumors were resected at week 4, and after 3 days, the mice were i.v. injected semiweekly with an anti-mouse IL-6R Ab (anti-IL-6R) or IgG2b, κ (IgG) for 3 weeks. The presented IVIS images indicate the tumor bioluminescence intensity at week 4, after tumorectomy (postsurgery) and the indicated immunotherapy for 2 weeks. MOCK indicated control (nonsurgery) mice (# 7 and # 8). Two xenograft mice (# 2 and # 24) never developed tumors even after the endpoint, probably due to strong immunosurveillance, and were excluded from the results (denoted by numbers in red). At week 4, tumors in shMCT-1 mice (# 15 and # 26) were palpable but small thus presented with no signal in the IVIS and still underwent tumorectomy. 4T1 xenografted nonsurgery (#9) and tumorectomy mice (# 13, # 18, # 22 and # 27) were dead at week 8 and week 2 postsurgery, respectively (denoted by crossed boxes), likely due to tumor aggressiveness or systemic inflammation in response to surgery and/or immunotherapy. The color bar indicated bioluminescence intensity. High bioluminescence intensity appeared as red, low intensity appeared as blue or dark purple. (B) Tumor mortality was monitored for 24 days. (C) Tumor metastasis rates were examined in mice without primary tumor removal (nonsurgery) and postsurgical distant metastasis or local recurrence under IgG2b, κ or IL-6R immunotherapy. (D-E) The amounts of CD163(+) M2 macrophages were detected by immunohistochemistry (D) and immunofluorescence (green, M2 macrophages; blue, nuclei) (E) (n = 6) in recurrent breast tumors. (F) The amounts of Foxp3(+) Tregs in relapsed breast tumors after immunotherapy (n = 6) were examined by immunohistochemistry. (G) TIMER 2.0 estimated the correlation of MCTS1 with Treg infiltration in the TCGA-BRCA cohort using the CIBERSORT-ABS algorithms and indicated as log2 transcripts per million (TPM). (H-I) Representative lung metastatic tumors (H) and lung nodules (as indicated by the arrow) (I) occurred in mice postsurgery and after IgG2b, κ (IgG) or anti-IL-6R treatment. (J) L*ymphangiogenesis effects* in metastatic tumor-draining lymph nodes (TDLNs) found in axillar lymph nodes (LNs) of nonsurgery scramble mice and normal axillary LNs (MOCK) (n = 2 ~ 10) were evaluated by Lyve-1 i*mmunostaining*. Metastatic TDLNs for remaining groups could not be detected macroscopically (denoted by ND: not detected). The symbol represents an individual sample with a middle horizontal bar at the mean ± SEM (D, F, I, J). Statistical impacts were analyzed by the log-rank (Mantel‒Cox) test (B), two-way ANOVA with Tukey (D, I) and Benjamini, Krieger and Yekutieli (F) post hoc analysis and a two-tailed unpaired Student's *t* test (J). *p < 0.05, **p < 0.01, ***p < 0.001.

Murine 4T1 tumor progression resembles advanced features of human TNBC metastasis to the lungs, liver, bone and brain 37, 48. To examine the potential of IL-6R immunotherapy against TNBC aggressiveness, the resulting 4T1 mammary tumors were surgically removed when the tumor size reached approximately 150 ~ 200 mm^3^ (Figure 4A). Starting on postsurgery day 3, anti-mouse IL-6R Ab (clone 15A7) and IgG2b, κ control Ab (clone LTF-2) were semiweekly administered to mice via i.v. injection for 3 weeks. The efficacy, safety, and tolerability of periodic IL-6R immunotherapy by i.v. administration have been tested in the previous pre-clinical models 49 and clinical trials 50. Representative bioluminescence images of mice exhibited the tumor intensity at week 4, postsurgery and the indicated immunotherapy for another 2 weeks (Figure 3A). In the 24-day postoperative period, shMCT-1 treatment sufficiently improved survival probability (Figure 4B), while the scramble cohort indeed exhibited a better survival rate in IL-6R immunotherapy than IgG2b, κ.

Even without resecting primary tumors (nonsurgery), the shMCT-1 group successfully inhibited metastasis (0%), but the scramble cohort still developed distant metastasis (66.8%) to the lungs, liver, or lymph nodes (Figure 4C). Postsurgical tumor relapse was also further inhibited by immunotherapy of IL-6R in the shMCT-1 group (12.5%) than in the scramble group (77.8%) when compared with IgG2b, κ treatment of scramble (100%) and shMCT-1 (55.6%) cohorts (Figure 4C). Unexpectedly, the surgical stress caused TNBC metastasis. For 2 ~ 3 weeks postsurgery, tumor metastasis to the lungs or the liver was promoted in both scramble (77.8%) and shMCT-1 (57.1%) conditions compared with nonsurgery conditions (66.8% and 0%, respectively) (Figure 4C). Importantly, anti-IL-6R immunotherapy inhibited postsurgical metastasis in the scramble group (33.3%) and further in the shMCT-1 group (27.3%) when compared with both groups were treated with IgG2b, κ (77.8% and 57.1%).

As evaluated by immunohistochemistry of the recurrent tumors, we further confirmed that anti-IL-6R reduced CD163(+) M2 macrophage (Figure 4D-E; Figure S3A) and Foxp3(+) Treg (Figure 4F; Figure S3B) infiltration, mostly in recurrent shMCT-1 tumors. Again, the TCGA-BRCA cohort study supported the conclusion that MCTS1 (MCT-1) activation is associated with Treg cell infusion (Figure 4G). As indicated by postsurgical breast cancer invasion of the lungs, the IgG2b, κ-treated scramble group showed many metastatic lung nodules, while fewer and smaller lung nodules occurred in the IgG2b, κ-treated shMCT-1 (Figure 4H-I). Metastatic lung nodules in both groups were significantly reduced by anti-IL-6R treatment (Figure 4H-I). Immunohistostaining of lymphatic vessel endothelial hyaluronan receptor 1 (Lyve-1) on tumor-draining lymph nodes (TDLNs) also indicated that the scramble group tumors promoted axillary lymph node metastasis with increased lymphangiogenesis when left unresected (Figure 4J). Notably, metastatic axillary TDLNs was undetectable across non-surgical shMCT-1 as well as IgG2b, κ- and anti-IL-6R-treated postsurgical groups (Figure 4J).

Our novel preclinical results prove that shMCT-1 alone sufficiently prevents TNBC immunity, metastasis, relapse and mortality. Combining IL-6R immunotherapy with the shMCT-1 effect could advance immunosurveillance that further avoids local recurrence and distant metastasis.

### IL-6R-based immunotherapy improves the immunity against TNBC

**Figure 5:**
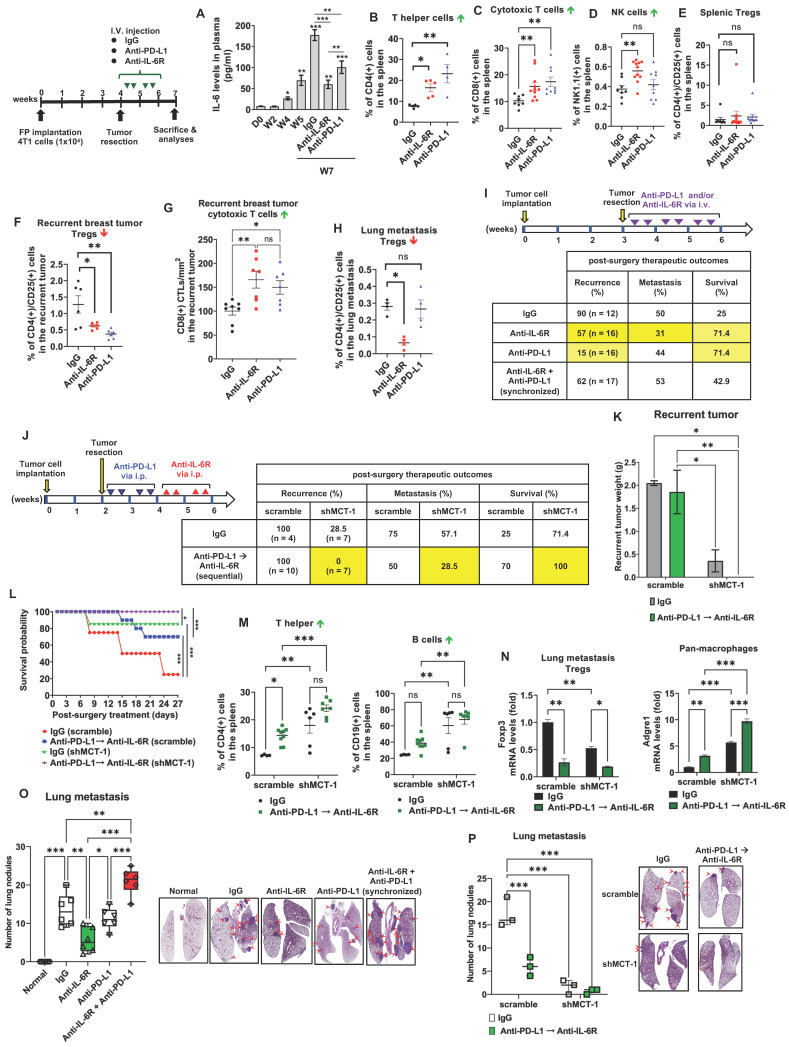
** Sequential immunotherapy of PD-L1 and IL-6R effectively inhibits TNBC aggressiveness with low MCT-1 expression.** 4T1 cells (1x10^4^) (scramble vs. shMCT-1) were orthotopically implanted into BALB/c mice. The resulting breast tumors were resected at week 4, and the mice were i.v. injected semiweekly with an anti-mouse IL-6R (n = 9), anti-mouse PD-L1 (n = 6) or IgG2b, κ (n = 5) Ab for another 2 weeks. Plasma IL-6 levels were analyzed during tumor progression and the indicated therapy using one-way ANOVA with the Newman‒Keuls test (A). At the endpoints (week 7), the splenic lymphocytes were analyzed for CD4(+) T helper cells (n = 4 ~ 5) (B), CD8(+) CTLs (n = 8 ~ 11) (C), NK cells (n = 8 ~ 11) (D) and CD4(+)/CD25(+) Tregs (n = 8 ~ 12) (E). Tregs (n = 6) (F) and CD8 CTLs (n = 7 ~ 8) (G) infiltration were measured in recurrent breast tumors. (H) Subsets of Tregs were examined in postsurgical lung metastasis (n = 4). All data were analyzed by one-way ANOVA with Dunnett's (B, D-F, H) or Sidak's test (G) except (C), which used the Kruskal‒Wallis test with Dunn's test. To examine postsurgical therapeutics, the primary breast tumors were resected (week 3). Subsequently, the mice were i.v. injected with IgG2b, anti-IL-6R, anti-PD-L1 Abs or synchronized immunotherapy (anti-IL-6R + anti-PD-L1) semiweekly for 3 weeks (n = 12 ~ 17) (I). Alternatively, the primary breast tumors were resected early (week 2) from the mice, and then IgG2b, κ treatment or sequential immunotherapy against PD-L1 and IL-6R (anti-PD-L1 → anti-IL-6R) was performed via i.p. injection for 2 weeks (J). Postsurgical and immunotherapeutic outcomes (I-J); tumor loads (K), two-way ANOVA with Tukey test; and survival rates (L), log-rank test were studied (n = 7). At the end point (week 7), splenic CD4(+) T helper and CD19(+) B cells were analyzed (M) using two-way ANOVA with the Tukey test. Adgre1 (F4/80 pan macrophages) and Foxp3 (Tregs) mRNA levels in lung metastatic breast tumors were measured (N), two-way ANOVA with Tukey test. Lung metastasis rates were evaluated in either monotherapy, synchronic (anti-IL-6R + anti-PD-L1) (n = 6) (O) or sequential (anti-PD-L1 → anti-IL-6R) (n = 3) (P) immunotherapy, one-way (O) and two-way (P) ANOVA followed by Tukey test. ns: not significant, *p < 0.05, **p < 0.01, ***p < 0.001.

Given that MCT-1 impacts on PD-L1 (CD274) (Figure 2D) and monotherapy of anti-PD-L1, atezolizumab, has been shown to prolong the survival of metastatic TNBC patients 51, we next asked whether immunotherapeutic efficacies of anti-IL-6R against TNBC expansion could be at least comparable to PD-L1 blockade. To this end, the resulting breast tumors from the scramble 4T1 cells-implanted mice were resected at week 4. Starting 3 days after surgery, the mice were i.v. injected semiweekly with anti-IL-6R Ab, anti-PD-L1 Ab (clone 10F.9G2) or IgG2b, κ for 2 weeks. As an indication of tumor invasion and severity, plasma IL-6 levels were continually elevated during tumor progression (Figure 5A). After postoperative tumor resection, plasma IL-6 was still induced prominently in the IgG2b, κ group. Escalation of circulating IL-6 levels has been previously reported to associate with systemic inflammation in cancer patients undergoing surgery 52, thus facilitating EMT and BCSCs expansion 33 and contributing to progressive metastasis 53. This was matched with increased distant metastasis after scramble and shMCT-1 groups underwent tumor resection (Figure 4C). The surge of circulating IL-6 levels could be greatly reduced by anti-IL-6R immunotherapy, and the extent of the reduction was much greater than anti-PD-L1 immunotherapy at the endpoint (week 7). Although IL-6 level was lower than IgG2b, κ treatment, we noticed that mice administered with anti-PD-L1 showed a gradual increase of plasma IL-6 level over the course of the treatment (Figure 5A). This is consistent to prior observations showing that patients with melanoma receiving immune checkpoints blockade have IL-6 upregulation 54, 55, which mediates immune-related adverse effects 56.

Examining splenic and tumor-infiltrating lymphocytes, anti-IL-6R promoted the amounts of splenic CD4(+) helper T cells (Figure 5B), CD8(+) CTLs (Figure 5C) and NK1.1(+) cells (Figure 5D; Figure S4A) but not CD4(+)/CD25(+) Tregs (Figure 5E, Figure S4B) as compared to isotype IgG treatment. However, anti-PD-L1 immunotherapy did not increase NK cells. Both anti-IL-6R and anti-PD-L1 significantly suppressed CD4(+)/CD25(+) Tregs (Figure 5F) but outscored IgG2b, κ in the infiltration of CD8(+) CTLs in the recurrent breast tumors (Figure 5G, Figure S4C), suggesting the effective anti-tumor immunity. Interestingly, only anti-IL-6R diminished Tregs in the metastatic lungs (Figure 5H; Figure S4B), comparing to isotype IgG-treated mice.

Reflecting on Figure 5A, we thus hypothesized that the combination of anti-IL-6R and shMCT-1 might limit IL-6 induction and consequently enhance therapeutic benefits of PD-L1 blockade in the metastatic recurrence TNBC. By testing this idea *in vitro* on BCSCs, the results showed that immunotherapy against IL-6R and/or PD-L1, especially in combination, effectively decreased 4T1 mammosphere formation (Figure S5A). Addition of anti-IL-6R to anti-PD-L1 further suppressed the mRNA levels of CSC markers, such as EpCAM (Figure S5B), Oct4 (Figure S5C) and Sox2 (Figure S5D), particularly in shMCT-1 background**.** CD24(-)/CD44(+) subpopulations in the scramble group (31.1%) were also reduced and they were further reduced in the shMCT-1 context (23.7%) upon combined IL-6R and PD-L1 immunotherapy compared with monotherapy (Figure S5E) when they compared with isotype IgG (MOCK) treatment (Figure S5F-I).

To examine the impact of immunotherapeutic BCSCs on macrophage polarity, Raw264.7 M0-like macrophages were treated with LPS and IL-4 to activate M1- and M2-like macrophage differentiation, respectively. The M1 macrophages expressed more iNOS, but the M2 macrophages presented more CD206 and IL-10 than the M0 macrophages (Figure S6A). CD68, CD80 and CD86 were expressed preferentially in M1 macrophages over M2 and M0 macrophages (Figure S6B). IL-10 and Arginase-1 (Arg-1) were enhanced in M2 macrophages more than in M1 and M0 macrophages (Figure S6C). Immunotherapeutic IL-6R and/or anti-PD-L1 blockades were pretreated 4T1 BCSCs (scramble vs. shMCT-1) before coculture with the polarized M2 macrophages in a Boyden chamber, which repolarized M2 toward an M1-like phenotype with increased CD80 (Figure S6D) and decreased M2 markers (CD163 and TGF-β) (Figure S6E-F), especially when encountering the coimmunotherapeutic BCSCs in the shMCT-1 group. Similarly, the immunotherapeutic BCSCs inhibited the invasiveness of M2 macrophages (Figure S6G). Hence, targeting multiple oncogenic points in BCSCs may advance immunosurveillance.

### Sequential immunotherapy against PD-L1 and IL-6R effectively inhibits TNBC metastasis and relapse

To further compare the immunotherapeutic outcomes, the primary 4T1 tumors were surgically removed in week 3, followed by immunotherapy of IL-6R and/or PD-L1 via i.v. injection semiweekly for 3 weeks (Figure 5I). Anti-PD-L1 showed the lowest tumor recurrence rate (15%) compared with anti-IL-6R (57%) and IgG 2b, κ (90%), but anti-IL-6R proved a better anti-metastasis outcome (31%) than anti-PD-L1 (44%) and IgG 2b, κ (50%) (Figure 5I). Both anti-PD-L1 and anti-IL-6R highly improved the survival results (71.4%) compared to IgG2b, κ control group (25%). However, synchronized coimmunotherapy (anti-IL-6R + anti-PD-L1) failed to advance the therapeutic outcomes compared with monotherapy, indicated by lower survival and higher metastasis rate (Figure 5I). This was likely due to enhanced systemic toxicity to multiple organs imposed by i.v. delivery of combined immunotherapy, as demonstrated in mouse models of cancer 57 and melanoma patients 58.

Therefore, we designed a sequential immunotherapy approach with i.p. administration (Figure 5J). Because i.p. injection of mAb has slow distribution in solid tumors 59, we used a 4-fold increase of anti-PD-L1 and anti-IL-6R doses (10 mg/kg) to expedite a stable distribution over the course of treatment. We expected this regimen to be safer but still clinically beneficial to improving TNBC recurrence and metastasis. The primary 4T1 tumors were resected early at week 2, and anti-PD-L1 was administered to the postsurgical mice for 2 weeks before anti-IL-6R treatment for another 2 weeks via intraperitoneal (i.p.) injection. At the indicated doses, the half-lives for i.p. anti-PD-L1 and anti-IL-6R are two 60 and four days 46, respectively, therefore we used semiweekly administration for both Abs to maintain uninterrupted IL-6R and PD-L1 blockade. Surprisingly, in shMCT-1 circumstances, sequential immunotherapy (anti-PD-L1 → anti-IL-6R) successfully prevented tumor relapse (0%) and metastasis (28.5%), achieved a disease-free *survival* advantage (100%) (Figure 5J) and diminished the recurrent tumor loads (Figure 5K). In the sequential immunotherapy, the scramble group still faced tumor remission (100%), postsurgery metastasis (50%) and lower survival outcome (70%) (Figure 5J). Consequently, shMCT-1 benefited and advanced the continuous immunotherapy regimen, sustaining survival probability (Figure 5L). At the endpoint (week 7), upon sequential PD-L1 and IL-6R immunotherapy, splenic lymphocytes of the shMCT-1 tumor-bearing mice increased more CD4(+) helper T cells and CD19(+) B cells (Figure 5M) but unchanged CD8(+) CTLs and NK cells (Figure S6H) when compared with those bearing scramble tumors and treated by isotype IgG. Sequential immunotherapy alongside shMCT-1 effect also significantly induced Adgre1 (F4/80 pan macrophages) and inhibited Foxp3 (Tregs) gene expression in postsurgical lung metastasis tumors (Figure 5N). In addition, the reduced lung nodules proved that anti-IL-6R and anti-PD-L1 monotherapies were equally efficient; however, synchronized immunotherapy (anti-IL-6 + anti-PD-L1) did not stop lung metastasis (Figure 5O). Differently, both sequential immunotherapy (anti-PD-L1 → anti-IL-6R) and shMCT-1 treatment efficiently prevented postsurgical tumor metastasis to the lungs (Figure 5P), particularly in combination of the sequential immunotherapy with shMCT-1 effect.

Accordingly, the earlier primary tumorectomy, delivery of combinational and chronological immunotherapy will halt tumor spreading and enhance TNBC therapeutics.

### Positive feedback interconnections of the MCT-1/IL-6/IL-6R/CXCL7/PD-L1 axis in TNBC cells

**Figure 6:**
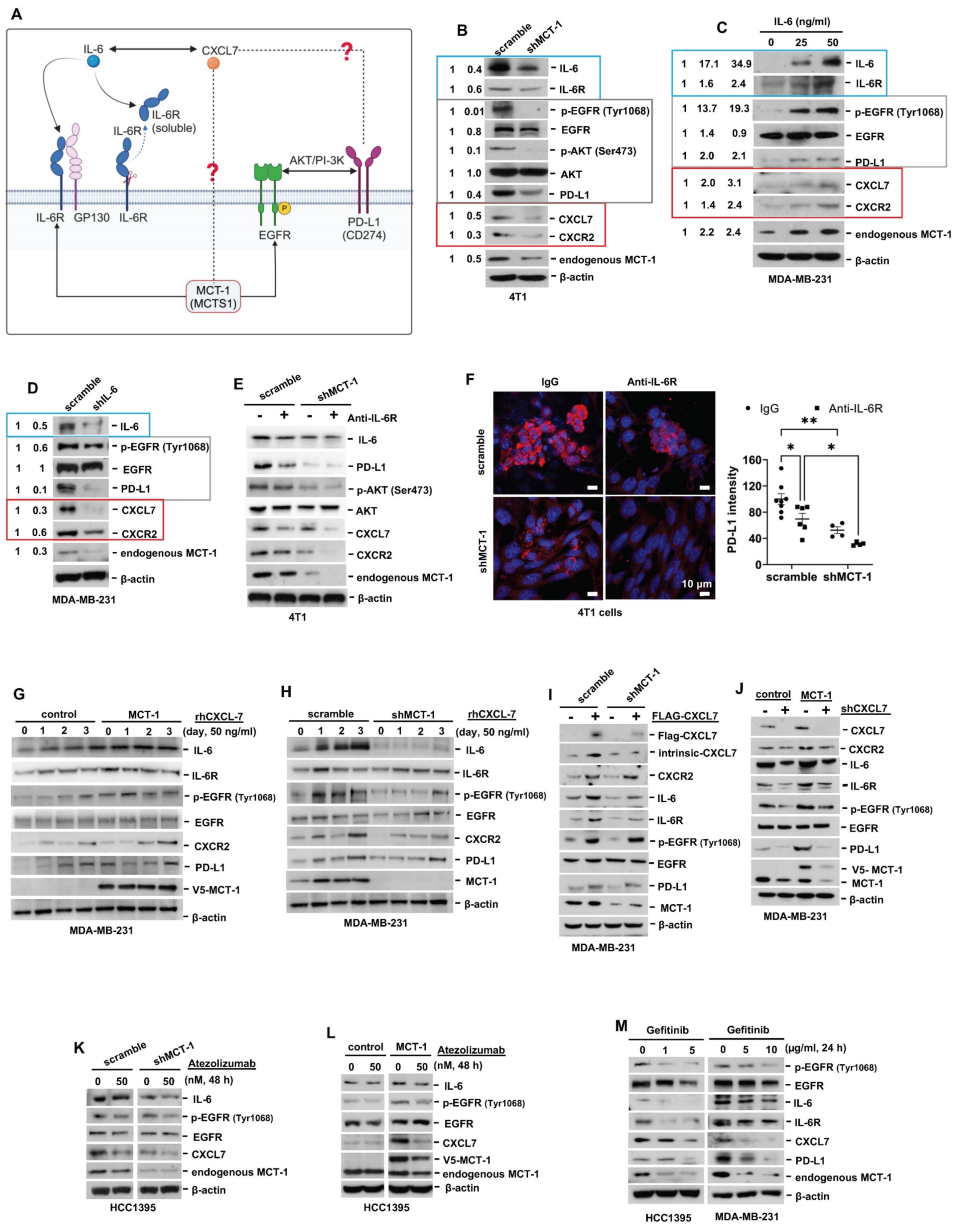
** Interconnections of MCT-1/IL-6/CXCL7/EGFR/PD-L1 signaling loops in TNBC cells.** (A) Schematic depicting working hypothesis with feedback loop connecting IL-6, CXCL7 and PD-L1 in the MCT-1 pathway. Schematic were created by BioRender. Composite shapes showing IL-6/IL-6R/gp130 were modified from “Tocilizumab Humanized Antibody Against IL-6R” template by BioRender. The indicated proteins were analyzed in 4T1 cells (scramble vs. shMCT-1) (B) and MDA-MB-231 (IV2-3) cells upon IL-6 stimulation (0 ~ 50 ng/ml) for 24 h (C) and IL-6 knockdown (scramble vs. shIL-6) (D). Cell lysates were immunoblotted with corresponding Abs. The relative protein level was normalized to the internal β-actin level and then compared with that in the scramble cells or IL-6 at 0 ng/ml (B-D). (E) The indicated proteins were analyzed in 4T1 cells without or with anti-mouse IL-6R (50 μg/ml) for 24 h. (F) Immunofluorescence of PD-L1 in 4T1 cells after IgG2b, κ (50 μg/ml) or anti-mouse IL-6R (50 μg/ml) for 24 h. Data are presented as the mean ± SEM (n = 4 ~ 7) and were analyzed by two-way ANOVA with the Tukey post hoc test. The indicated proteins were analyzed in MDA-MB-231 (IV2-3) cells with or without MCT-1 overexpression (V5-tagged) (control vs. MCT-1) (G) or depletion (H) under rhCXCL7 treatment (50 ng/ml) for 0 ~ 3 days. (I) FLAG-tagged CXCL7 and (J) CXCL7 silencing (shCXCL7) were introduced to MDA-MB-231 (IV2-3) cells and the indicated proteins were assayed. In HCC1395 cells with or without MCT-1 overexpression (K) or depletion (L), the indicated protein levels were examined upon atezolizumab (humanized anti-PD-L1) (50 nM) treatment for 48 h. (M) HCC1395 and MDA-MB-231 cells were exposed to different doses of gefitinib (IRESSA) (0 ~ 10 µg/ml) and immunoblotted. *p < 0.05, **p < 0.01.

CXCL7 and IL-6 mediate the positive feedforward interaction between mesenchymal stem cells (MSCs) and tumor cells 61, 62. EGFR induces PD-L1 via the IL-6/JAK1/STAT3 pathway 63, which reciprocally drives PD-L1 phosphorylation (Tyr112) to evade cancer immunosurveillance 64. PD-L1 activates PI-3K/AKT pathway, while activated PI-3K/AKT can also increase PD-L1 expression 65. Given that MCT-1 has been shown to induce EGFR 31 and IL-6/IL-6R/gp130 33, we thus hypothesized that CXCL7 is the hub connecting IL-6 and PD-L1 in the MCT-1 pathway, as modeled in Figure 6A.

To identify the signaling pathways underlying the efficacy of combinatorial immunotherapy (anti-PD-L1 → anti-IL-6R) in preventing recurrence and metastasis, we questioned the involvement of CXCL7 within MCT-1 oncogenic pathway. Depleting MCT-1 in 4T1 cells (p53-null, wild-type BRCA1/2) effectively decreased PD-L1, IL-6/IL-6R and p-EGFR (Tyr1068) (Figure 6B). This was parallel to what we found via transcriptomics (Figure 2C-D). As expected, reduced PD-L1 could be marked by inactivated p-AKT (Ser473) (Figure 6B). Here, we demonstrated for the first time that shMCT-1 reduced CXCL7 and one of its receptors, CXCR2, (C-X-C chemokine receptor 2, also known as interleukin-8 receptor beta, IL8RB). Consistently, reduction of PD-L1, CXCL7/CXCR2, IL-6/IL-6R and p-EGFR (Tyr1068) by shMCT-1 was observed in human TNBC HCC1395 cells (mutated p53 and BRCA1, derived from early stage TNBC) (Figure S7A). When MCT-1 was overexpressed (V5-tagged, indicated as V5-MCT-1), PD-L1 and CXCL7/CXCR2 were induced with concordant increases in IL-6/IL-6R and p-EGFR (Tyr1068) (Figure S7B). At this point, we confirmed that CXCL7/CXCR2 and PD-L1 are regulated by MCT-1 in TNBC cells in both human and mouse TNBC cell lines.

We next asked how IL-6 affected MCT-1, CXCL7 and PD-L1. To this end, IL-6 was used to treat TNBC MDA-MB-231 (IV2-3) cells (mutated p53 and BRCA2, a highly invasive subline derived from two rounds of *in vivo* selection of lung metastasis) 66. IL-6 not only enriched PD-L1 and CXCL7/CXCR2, but also induced MCT-1, IL-6R and p-EGFR (Tyr1068) in a dose-dependent manner (Figure 6C). Conversely, silencing IL-6 (shIL-6) reduced p-EGFR (Tyr1068), PD-L1, CXCL7/CXCR2 and MCT-1 levels (Figure 6D). In 4T1 scramble cells, inhibition of PD-L1 and CXCL7 could be replicated using anti-IL-6R as compared to IgG2b, κ (Figure 6E), but followed by minor effects on CXCR2 and MCT-1 levels. However, we noted that shMCT-1 further amplified reductions on p-AKT (Ser473), PD-L1, CXCL7/CXCR2 and MCT-1 levels exerted by anti-IL6R (Figure 6E). Immunostaining further indicated that anti-IL-6R combined with shMCT-1 dramatically lowered PD-L1 expression compared with scramble cells and those treated with IgG2b, κ (Figure 6F). These results verified our notion that inflammatory signals from IL-6 upregulates MCT-1/CXCL7 and PD-L1. Moreover, we identified that IL-6R immunotherapy alongside shMCT-1 treatment additively decreased CXCL7/CXCR2 and PD-L1, which possibly explained why combined immunotherapy of anti-PD-L1 and anti-IL-6R worked better when MCT-1 expression was low.

To test this idea, we did two approaches. The first approach was to understand the impact of MCT-1/CXCL7 on PD-L1. In MDA-MB-231 (IV2-3) cells, MCT-1 overexpression induced IL-6, p-EGFR (Tyr1068) and PD-L1 (Figure 6G). We also found that recombinant human CXCL7 (rhCXCL7) treatment increased IL-6/IL-6R/p-EGFR (Tyr1068), CXCR2, PD-L1 and ectopic MCT-1 (V5-MCT-1) levels in a time-dependent manner (Figure 6G). Additionally, rhCXCL7 counteracted the effect of shMCT-1, which restored IL-6/IL-6R, p-EGFR (Tyr1068), CXCR2 and PD-L1 levels (Figure 6H). Similar results were identified while stimulating HCC1395 cells with rhCXCL7 (Figure S7C-D), highlighting that MCT-1/CXCL7 elevated PD-L1 expression. Introducing FLAG-tagged CXCL7 (FLAG-CXCL7) in MDA-MB-231 (IV2-3) cells antagonized the shMCT-1 effects and restored CXCR2, IL-6/IL-6, p-EGFR (Tyr1068), PD-L1 and MCT-1 levels (Figure 6I). Conversely, shCXCL7 caused reductions in intrinsic and extrinsic MCT-1, thus diminishing the promotive effects of MCT-1 on CXCL7/CXCR2, IL-6/IL-6R, p-EGFR (Tyr1068) and PD-L1 (Figure 6J). Taken together with Figure 6B and Figure S7A-S7B, these results indicated that CXCL7 enriches MCT-1 in a feedback loop, where CXCL7 amplifies MCT-1 signaling to generate more CXCL7. Chemokine signaling such as CXCL12 and CXCR1/CXCR2 has been shown to repulse infiltration of T cells and neutrophils if present at high concentrations 67, 68, restricting immune response at tissue microenvironment. This could partially explain why the abundance of MCT-1 led to lack of response to immunotherapy.

On the second approach, we employed atezolizumab to reveal the effect of PD-L1 blockade in the MCT-1 pathway. In parallel with anti-IL-6R effects (Figure 6E), atezolizumab broadened the ability of shMCT-1 to diminish CXCL7 and p-EGFR (Tyr1068) (Figure 6K). Atezolizumab also repressed IL-6, CXCL7 and p-EGFR (Tyr1068) levels induced by MCT-1 overexpression (Figure 6L). We further asked whether these effects were consistent if PD-L1 expression was impaired by deactivation of EGFR. By employing pharmacological inhibitor of EGFR, gefitinib (IRESSA) in HCC1395 and MDA-MB-231 (IV2-3) cell lines (Figure 6M), we observed that IL-6/IL-6R, CXCL7 and MCT-1 levels were steadily reduced in a dose-dependent manner following PD-L1 reduction. Altogether, these results prove that EGFR and PD-L1 mutually promote the MCT-1/IL-6/CXCL7 pathway in the TNBC cellular milieu.

Collectively, the positive feedback loops existed within MCT-1/IL-6/IL-6R/CXCL7/PD-L1 pathway reveal the underlying mechanism by which sequential immunotherapy of PD-L1 and IL-6R could powerfully inhibit TNBC aggressiveness, specifically in the shMCT-1 context.

### Enrichments of MCT-1, IL-6/IL-6R, CXCL7 and PD-L1 predict poor prognosis of breast cancer

**Figure 7:**
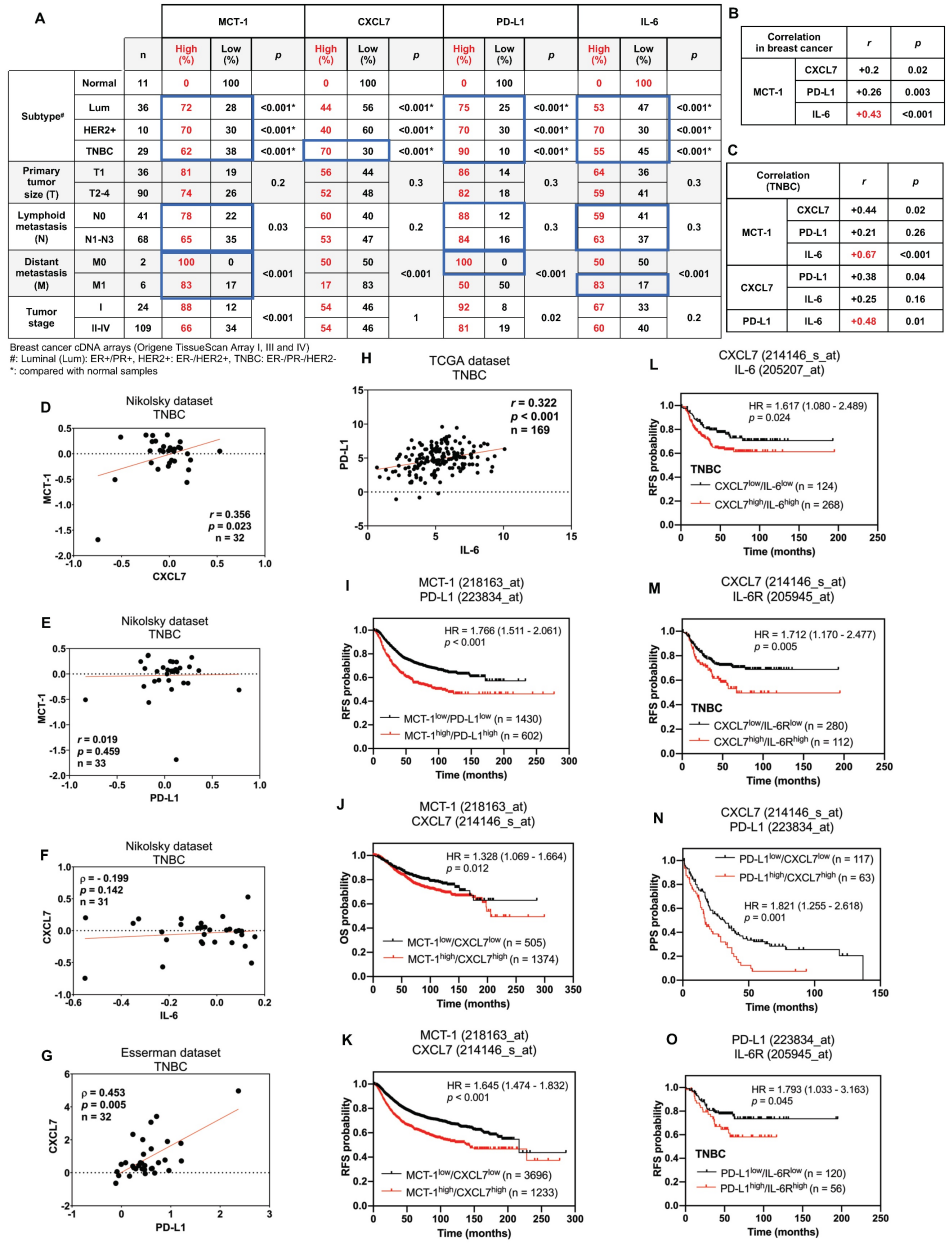
** Enrichments of MCT-1, IL-6/IL-6R, CXCL7 and PD-L1 predict poor prognosis in breast cancer patients.** The relative MCT-1, CXCL7, PD-L1 and IL-6 mRNA levels were quantified by qRT‒PCR using Origene TissueScan cDNA Arrays (I, III and IV) after normalization to β-actin mRNA levels. High expression represents a ≥ 1.5-fold change, while low expression represents a < 1.5-fold difference in breast cancer samples (n = 133) when compared with the average level of the indicated gene in normal breast samples (n = 11). The results were stratified by molecular subtype, TNM classification and tumor stage. The chi-squared test indicates a statistical significance between pairwise comparisons (A). The *r* coefficient indicates the association between two genes in total breast cancers (n = 133) (B) and in TNBCs (n = 29) (C). The Oncomine (D-G) or cBioPortal (H) database was used to assess the clinical relations of MCT-1 vs. CXCL7 (D), MCT-1 vs. PD-L1 (E), CXCL7 vs. IL-6 (F), CXCL7 vs. PD-L1 (G) and PD-L1 vs. IL-6 (H) in TNBC patients. Pearson's (*r*) or Spearman's (⍴) correlation indicates the strength of connection between two genes (B-H). Regression line was shown in red solid line. mRNA expression is reported as fold change (D-G) or RSEM (batch normalized from Illumina HiSeq_RNASeqV2) (log2) (H). Kaplan‒Meier Plotter was used to assess the correlation of patient survival with gene expression, and the hazard ratio (HR) (95% confidence interval) and *p*-value were calculated by univariate (I-K, N) or multivariate (L, M, O) Cox regression analysis. The probabilities of RFS (I, K-M, O), OS (J) and PPS (N) linked to MCT-1/PD-L1 (I), MCT-1/CXCL7 (J, K), CXCL7/IL-6 (L), CXCL7/IL-6R (M), PD-L1/CXCL7 (N) and PD-L1/IL-6R (O) expression patterns were analyzed. Probe IDs of corresponding genes were indicated on the top of the survival plot.

As we evaluated the clinical relevance of MCT-1, CXCL7, PD-L1 and IL-6 using the cDNA arrays of breast cancer and normal breast biopsies (OriGene TissueScan), we found that MCT-1, PD-L1 and IL-6 mRNAs were enriched in luminal, HER2(+) carcinomas and TNBCs, except for CXCL7, which was only induced in TNBCs (Figure 7A). Both primary and lymph node metastasis (N1-N3) of breast cancers promoted MCT-1, PD-L1 and IL-6 expression, while MCT-1 and IL-6 were upregulated in another distant metastasis (M1), supporting their metastatic roles.

In overall breast cancers, MCT-1 expression was more positively associated with IL-6 (*r* = 0.43, p < 0.001) than with CXCL7 (*r* = 0.2, p = 0.02) and PD-L1 (*r* = 0.26, p = 0.003) (Figure 7B). In TNBCs, MCT-1 expression level also significantly connected with IL-6 (*r* = 0.67, p < 0.001) more than with CXCL7 (*r* = 0.44, p = 0.02) but negligibly connected with PD-L1 (*r* = 0.21, p = 0.26) (Figure 7C). Likewise, TNBCs revealed more positive relations between PD-L1 and IL-6 (*r* = 0.48, p = 0.01) than between CXCL7 and PD-L1 (*r* = 0.38, p = 0.04) while no relation was found between CXCL7 and IL-6 (*r* = 0.25, p = 0.16) (Figure 7C). We next performed Oncomine 69 and cBioPortal 70 database mining to verify these clinical correlations in other datasets. We found that TNBCs exhibited positive correlation between MCT-1 and CXCL7 (Nikolsky, *r* = 0.356, p = 0.023) (Figure 7D). This correlation between MCT-1 and CXCL7 was not present in luminal (Figure S8A-B) but present in HER2(-) subtypes (Nikolsky, *r* = 0.383, p < 0.001) (Figure S8C). Consistent to Figure 6C, MCT-1 did not significantly associate with PD-L1 in both TNBCs (Nikolsky, *r* = 0.019, p = 0.459) (Figure 7E) and HER2(+) subtype (Nikolsky, ⍴ = -0.036, p = 0.429) (Figure S8D). Of note, ER and/or PR status affected clinical association between MCT-1 and PD-L1 (Nikolsky, ⍴ = 0.415, p < 0.001) (Figure S8E). The same pattern was observed between CXCL7 and IL-6 as we found that TNBCs (Nikolsky, ⍴ = - 0.199, p = 0.142) (Figure 7F) and HER2(+) subtype (Nikolsky, *r* = 0.186, p = 0.193) (Figure S8F) did not indicate any association unless the case with hormone status was considered (Nikolsky, ⍴ = 0.382, p < 0.001) (Figure S8G). Like what we confirmed in Figure 6C, CXCL7 and PD-L1 showed positive correlation in TNBCs (Esserman, ⍴ = 0.453, p = 0.005) (Figure 7G), but not in luminal and HER2(+) subtypes (Figure S8H-I). We also verified positive correlation between expression of PD-L1 and IL-6 in both TNBC (Figure 7H) (TCGA, *r* = 0.322, p < 0.001) and luminal subtype (Figure S8J) (TCGA, *r* = 0.202, p < 0.001) while no apparent association was found in HER2(+) subtype (Figure S8K).

The Kaplan‒Meier Plotter database containing pooled datasets of breast cancers adjusted with univariate or multivariate Cox regression was further analyzed the clinical relevance. Patients with MCT-1^high^/PD-L1^high^ expression showed lower recurrence-free survival (RFS) than patients with MCT-1^low^/PD-L1^low^ expression (HR 1.766 [95% CI 1.511-2.061]; p < 0.001) (Figure 7I). MCT-1^high^/CXCL7^high^ expression was associated with poorer overall survival (OS) (HR 1.328 [95% CI 1.069-1.664]; p = 0.012) (Figure 7J) and RFS (HR 1.645 [95% CI 1.474-1.832]; p < 0.001) (Figure 7K). CXCL7^high^/IL-6^high^ expression also exhibited reduced RFS in TNBC patients (HR 1.617 [95% CI 1.080-2.489]; p = 0.024) (Figure 7L). Also, CXCL7^high^/IL-6R^high^ expression exhibited poorer RFS in patients with TNBC (HR 1.712 [95% CI 1.170-2.477]; p = 0.005) (Figure 7M). Similarly, PD-L1^high^/CXCL7^high^ expression revealed lower postprogression survival (PPS) than PD-L1^low^/CXCL7^low^ expression (HR 1.821 [95% CI 1.255-2.618]; p = 0.001) (Figure 7N). PD-L1^high^/IL-6R^high^ expression also had significant impact on RFS of TNBC patients (HR 1.793 [95% CI 1.033-3.163]; p = 0.045) (Figure 7O). Therefore, enhancements of MCT-1, IL-6/IL-6R, CXCL7 and PD-L1 expression are risk factors for predicting breast cancer violence and unfavorable clinical outcomes.

## Discussion

We demonstrate for the first time that interconnections of MCT-1-enhanced IL-6/IL-6R, CXCL7/CXCR2 and p-EGFR/PD-L1 exist in TNBC cells (Figure 6, Figure S7), which promote TNBC stemness, immunity and aggression. IL-6 acts as an autocrine that enhances the MCT-1 level, forming a positive feedforward (MCT-1 - IL-6 - IL-6R - MCT-1) through IL-6R activation. Additionally, MCT-1 enhances CXCL7/CXCR2 levels. Furthermore, CXCL7 acts on CXCR2 in an autocrine manner and induces the MCT-1 level, forming another positive feedback loop (MCT-1 - CXCL7 - CXCR2 - MCT-1) through CXCR2 activation. The two feedforward loops communicate because IL-6 enhances CXCL7/CXCR2 levels, and CXCL7 consecutively increases the IL-6/IL-6R levels (IL-6/IL-6R - CXCL7/CXCR2 - IL-6/IL-6R). Importantly, both IL-6 and CXCL7 activate EGFR, which reciprocally induces more IL-6/IL-6R, CXCL7/CXCR2 and MCT-1, forming another positive feedback loop (MCT-1 - IL-6/IL-6R - CXCL7/CXCR2 - pEGFR - MCT-1). We noticed that PD-L1 amplifies these networks by activating the IL-6-CXCL7-EGFR pathway. The above molecules are prognostic markers for poor unfavorable survival in breast cancer (Figure 7).

Our preclinical studies suggested that systematically targeting the MCT-1/IL-6/IL-6R/CXCL7/PD-L1 nexus could be a new medical opportunity that would benefit long-term cancer control and disease-free survival. Depletion of MCT-1 alone sufficiently downregulated inflammatory and proliferation pathways (Figure 2-3), which may prevent high risks of TNBC recurrence and metastasis (Figure 4). IL-6R immunotherapy plus MCT-1 knockdown effectively decreased the immunosuppressive TME, better supporting the prevention of primary and secondary tumor spreading than monotherapy (Figure 4). Although TNBC surgery faces challenges of recurrence and metastasis due to postoperative systemic inflammation (Figure 5A), silencing MCT-1 or immunotherapeutic IL-6R proficiently inhibited these side effects (Figure 4C, 5N), while anti-PD-L1 mainly prevented local relapse (Figure 5I). Anti-IL-6R can also improve anti-PD-L1 efficacy, presumably, by lowering plasma IL-6 levels (Figure 5A) secreted by many cell types in TME during TNBC progression and decreasing Treg accumulation in metastatic lungs (Figure 5H). Blocking IL-6/IL-6R pathway in TNBCs may help overcome the immune evasion of anti-PD-1/PD-L1 immunotherapy, which lacks PD-L1 or resists PD-1/PD-L1 blockades. We demonstrated that using anti-IL-6R alone or sequential immunotherapy of PD-L1 and IL-6R profoundly abolished TNBC violence and reprogrammed the plasticity of immune cells (Figure 5J-N, P). Consistently, combined immunotherapy against IL-6R and PD-L1 counteracted TNBC stemness (Figure S5A-E) that led to suppressing the polarity and invasiveness of M2 macrophages (Figure S6D-G)* in vitro*, probably stopping BCSCs escape from immune surveillance. Our pre-clinical models did not allow comprehensive dissection of which cell types in TME respond to IL-6R blockade over the course of combined immunotherapy. Future studies with lineage-specific deletion of IL-6R are needed to investigate the efficacy and the mechanisms underlying this combination.

A key finding of this study was that sequential immunotherapy (anti-PD-L1 → anti-IL-6R) showed a very potent anti-recurrence effect in shMCT-1 (Figure 5J-L). Despite improved survival and lower metastasis rate (Figure 5J, L, P), we did not identify any changes in local recurrence in scramble mice following anti-PD-L1 → anti-IL-6R (Figure 5J). This suggests critical clinical implication that anti-PD-L1 → anti-IL-6R is likely more effective in TNBC patients with MCT-1^low^ rather than those with MCT-1^high^. Reflecting to our earlier work 33, anti-PD-L1 → anti-IL-6R would benefit approximately 29.3% of TNBC patients who were classified as MCT-1^low^. Anti-PD-L1 → anti-IL-6R could also offer meaningful improvement in 29.6% and 32.4% MCT-1^low^ TNBC patients with invasive (IDC) stage and lymphatic metastasis (N1-N3), respectively (Figure 1J). Herein, although TNBC patients with MCT-1^low^ have relatively low prevalence, this study underscores a new perspective that anti-PD-L1 → anti-IL-6R can be favorable for early stage TNBC, which is often attributed with smaller tumors (Figure 5K) and lymph node involvement (Figure 1J) as evidenced by MCT-1 depletion.

It is important to note that the superiority of sequential (anti-PD-L1 → anti-IL-6R) (Figure 5J) over the synchronous immunotherapy (anti-IL-6R + anti-PD-L1) (Figure 5I) in TNBC was not conclusive because it was not powered for efficacy comparisons between arms. Better therapeutic outcomes observed from sequential immunotherapy could also be stemmed from two factors: (1) increased dosing regimens and (2) different route of administration. Because anti-PD-L1 has comparable pharmacokinetics across low and high dosing regimens 71 while anti-IL-6R has dose-proportional pharmacokinetics 72, it raises the possibility that anti-IL-6R dose is a determinant factor for clinical benefits for TNBC immunotherapy. Dosing regimens of anti-IL-6R can thus be leveraged to improve therapeutic outcomes when used in combination with anti-PD-L1. Moreover, local administration that mitigated the toxicity of mAbs can also contribute to the outcomes of combined immunotherapy. For instance, synchronous immunotherapy of anti-PD-L1 and anti-IL-6R via i.p. has been reported to reduce pancreatic tumor progression and improve survival in mice without apparent signs of toxicity 18. In contrast, sequential immunotherapy of atezolizumab and tocilizumab via i.v. infusion can lead to adverse events such as elevated aspartate aminotransferase, resulting in insignificant extension of survival of metastatic urothelial carcinoma patients as compared with atezolizumab monotherapy 73. Further research is warranted to determine best regimens for treating TNBC with combined IL-6R and PD-L1 immunotherapies.

CXCL7 elevation predicts relapse and poorer clinical outcome of invasive colorectal cancer 74. CXCL7 induces VEGF-C and VEGF-D in breast cancer cells 75, which mediate angiogenesis and lymphangiogenesis in the primary tumor to provide routes facilitating tumor dissemination. CXCL7 promotes the metastatic potential of TNBC 16, lung cancer 76 and cholangiocarcinoma 77. Targeting the CXCL7/CXCR2 pathway with a CXCR2 antagonist (SB265610) has been reported to decrease lung metastasis prevalence and reinforce CD8(+) CTLs in the spontaneous mammary tumors of MMTV-PyMT mice in chronic circadian disruption 78. CXCL7-based immunotherapy has been found to prevent TNBC progression and metastasis in mice 16. shMCT-1 reduced CXCL7/CXCR2 (Figure 6B, Figure S7A), implying how shMCT-1 stopped TNBC invasion to the lungs and lymph nodes (Figure 4H-J). Inhibition of IL-6R, PD-L1 or EGFR resulted in CXCL7 decline (Figure 6E, 6K-M), and shCXCL7 decreased MCT-1, IL-6/IL-6R, p-EGFR and PD-L1 (Figure 6J), which may potentiate therapeutic efficacy against tumor spreading via lymphatic circulation. EGFR antagonists combined with immunotherapeutic IL-6/IL-6R, PD-L1 or CXCL7/CXCR2 blockade may advance TNBC treatment. In agreement with this notion, the previous study has reported that inhibition of the PD-1/PD-L1 axis increases CD8(+) CTLs and inhibits rhabdomyosarcoma in CXCR2-deficient mice 79.

IL-6 and CXCL7 also facilitate the interplay between MSCs and BCSCs 62. BCSCs secrete IL-6, which induces MSCs to produce CXCL7, increasing BCSCs and metastaticity. Moreover, IL-1β induces CXCL7, which accelerates clear cell renal cell carcinoma growth in mice, is inhibited by anti-CXCL7 Abs 80. Our results also prove that IL-6/IL-6R crosstalk with CXCL7 was observed in TNBC cells (Figure 6C-D, 6G-K), and their enrichments are independent of prognostic factors in patients with TNBC (Figure 7L-M).

We reported that MCT-1 activates EGFR/AKT signaling and decreases tumor suppressors such as PTEN, p53 and miR-34a in cancer cells 29, 30, 33. Notch-1 mediates PTEN inhibition, which results in ERK1/2 activation, HER2(+) breast cancer cell proliferation and BCSC survival 81. Similarly, upregulation of miR-34 inhibits breast tumor progression and metastasis 82. Both PTEN and miR-34a functionally suppress PD-L1 83, 84, whereas the AKT-mTOR pathway promotes PD-L1 65. PTEN also suppresses NF-κB-induced cytokines and growth factors 85, which affect accumulation of macrophages, neutrophils and Tregs in tumors. The p53 status shapes the tumor immune landscape 86. Normal p53 induces miR-34a that targets the 3' untranslated region of PD-L1 87, whereas mutant p53 in human lung cancer enriches *PD-L1* 88, 89. Therefore, through deregulation of oncogenic kinases and tumor suppressors, MCT-1 can promote PD-L1 to supersede immune checkpoint control.

## Conclusion

We found that systematically and sequentially target the MCT-1/IL-6/IL-6R/CXCL7/PD-L1 axis by immunotherapies and antagonists can fight against TNBC heterogeneity, immunity and aggressiveness, which will have therapeutic benefits to improve patient survival.

## Methods

### RNA sequencing analysis

Total mRNA samples were isolated by a RNeasy Mini Kit (Qiagen, Germantown, MD) and quantified using the Qubit assay (Thermo Fisher Scientific, Waltham, MA). RNA quality control was performed using the Fragment analyzer. A Universal Plus mRNA-Seq kit (NuGEN/Tecan, Redwood City, CA) was used to generate RNA-Seq libraries derived from poly(A)-selected RNA according to the manufacturer's instructions. Universal Plus mRNA-Seq libraries contain dual (i7 and i5) 8 bp barcodes and an 8 bp unique molecular identifier (UMI), which enable deep multiplexing of NGS sequencing samples and accurate quantification of PCR duplication levels. The AnyDeplete workflow was used to remove unwanted ribosomal and globin transcripts before PCR enrichment. All library preparations were performed using the Biomek i7 laboratory automation system (Beckman Coulter, Mannedorf, Switzerland).

Pooled libraries were sequenced on an Illumina NovaSeq 6000 platform (Illumina, San Diego, CA) by using a paired-end 100 base pair run configuration. Raw FASTQ files were trimmed using cutadapt (v1.18) 90, and the trimmed reads shorter than 20 base pairs were removed. FastQC (v0.11.8) was used to generate prealignment QC metrics 91. STAR (v2.7.0d) was used to index and align reads to release 19 of the gencode mm10 genome and gene annotation 92. Filtering of low-expressed genes and normalization were performed using RSEM gene counts, defined as having 1 or fewer counts per million in at least two samples. To generate normalized sample-level data, filtered gene counts were TMM normalized using edgeR, followed by conversion to log counts per million with edgeR::cpm 93. DEGs analyzed by edgeR were genes with adjusted *p*-value < 0.05 identified by Fisher's exact test. Normalized and processed data alongside pairwise DEGs are provided in Table S3. These processing steps followed the MoTrPAC Consortium pipeline for RNA Sequencing 94. Gene Ontology (GO) functional annotations of overlapping transcripts among pairwise DEGs (Table S4) was performed using the Protein Analysis Through Evolutionary Relationships (PANTHER) system 95.

### Microarray and GSEA

Total mRNA samples were isolated from the 4T1 cells using the RNeasy Mini Kit (Qiagen, Germantown, MD), reversely transcribed by the GeneChip WT PLUS Reagent Kit (Thermo Fisher Scientific, Waltham, MA) and assessed with the Clariom D Array, mouse (Thermo Fisher Scientific). DEGs and hierarchical clustering were analyzed using Partek Genomic Suite 7.0 (Partek Incorporated, St. Louis, MO). DEGs were genes with fold change at least 1.2 and *p*-value < 0.05 calculated by ANOVA (Table S1).

For GSEA, normalized and processed transcripts of shMCT-1 vs. scramble (Figure 2B) and shMCT-1 vs. scramble (anti-IL6R vs. IgG2b, κ) (Figure 3C-E) were used as the input in GSEA 4.1 96. Gene set permutation was performed for evaluating the statistical significance of the enrichment score. Hallmark (H) gene sets in the Molecular Signatures Database (MSigDB) Human collections curated by GSEA was studied. Core genes (Table S2, S5) that contributed to the gene set's enrichment score were identified from the leading-edge subsets. Chord diagram visualizing connection between selected GSEA pathways and the log2 fold change of the core genes was generated using SRplot 97.

### Tumor progression, metastasis and recurrence

Six-week-old female BALB/c mice were obtained from the National Laboratory Animal Center (Taipei, Taiwan) and approved by the Animal Use Protocol (NHRI-IACUC-108026-A). 4T1 cells (1x10^3^ ~ 5x10^4^) bearing a pcDNA3.1-luciferase reporter were implanted in the 4th mammary fat pad. Tumor intensity was monitored after intraperitoneal (i.p.) injection of luciferin (150 mg/kg) (PerkinElmer, Waltham, MA) and detected with a Xenogen IVIS 200 bioluminescence system (Caliper Life Sciences, Hopkinton, MA). Tumor volumes were measured as previously described 33. Metastatic TDLNs were defined macroscopically as axillary LNs with enlargement ≥ 10 mm in diameter and collected when the mice were moribund as per protocol's specification. Axillary LNs serve as surrogate of TDLNs lymphangiogenesis because inguinal LNs within 4th mammary fat pad (tumor implantation site) drain to the axilla.

To evaluate postsurgical therapeutics, 4T1 cells (1x10^4^) were orthotopically injected. When the tumor volumes reached approximately 150-200 mm^3^ (weeks 3 ~ 4), the primary tumors were surgically removed and confirmed with the IVIS system. Subsequently, the mice were i.v. injected with an anti-mouse PD-L1 Ab (2.5 mg/kg) (clone 10F.9G2) (Bio X Cell, West Lebanon, NH) and anti-mouse IL-6R Ab (2.5 mg/kg) (clone 15A7) (Bio X Cell) alone or in combination, semiweekly for 3 weeks.

To study sequential immunotherapy, the primary 4T1 tumors were resected earlier (week 2) and then intraperitoneally (i.p.) injected with anti-mouse PD-L1 (10 mg/kg) (Bio X Cell) semiweekly for 2 weeks followed by anti-mouse IL-6R (10 mg/kg) (Bio X Cell) immunotherapy for another 2 weeks. The control group was treated with IgG2b, κ (clone LTF-2) (10 mg/kg) (Bio X Cell). At week 7, tumors were collected for further analysis. Tumor relapse, metastasis and mortality were determined. Tumor, spleen, and metastatic lung tissues were collected for immunohistochemistry (the detailed protocols are provided in Supplementary Methods), immunophenotyping using flow cytometry (the detailed protocols are provided in Supplementary Methods) and quantitative RT-PCR (the primer list is provided in the supplementary methods.

### Clinical study

Proportion of TNBC patients with protein expression levels of MCT-1 (Figure 1J) was based upon our previous work generated by staining human breast tissue arrays containing TNBC cases (BR1503c, BR953 and BRN801a from US Biomax, Rockville, MD; BRC964 from Pantomics, Richmond, CA) and normal breast tissue (Hbre-Duc052Bch-01, SOBC, Shanghai, China) with MCT-1 Abs (1:200, GTX117793, GeneTex, Irvine, CA) 33. Following histological quantification, the relative intensity of MCT-1 was classified into absent (-) and minor (1 +) as the cohort with low MCT-1 and distinct (+ 2) and strong (+ 3) as high MCT-1 cohort.

The indicated gene expression levels (Figure 7A) were quantified using TissueScan Breast Cancer Tissue cDNA Arrays I (BRCT101), III (BRCT103) and IV (BRCT104) (OriGene Technologies, Inc., Rockville, MD). This cDNA arrays cover samples from normal breast tissue (7.6%), primary breast tumor (88.9%), and recurrent breast tumor (3.5%) with various stage groups, TNM stages and molecular subtypes. Neoadjuvant therapy-related information was not available. Relative mRNA levels were calculated as follows: ΔΔCT = ΔCt cancer - ΔCt normal tissue. The fold change in the expression of each gene was calculated using the 2^-ΔΔCT^ method. For cDNA arrays, patient cohorts were stratified into high (≥ 1.5-fold) and low (< 1.5-fold) expression groups based on the median level of indicated gene in normal breast tissue. Clinical attributes pertaining to the tissue and cDNA arrays were provided by the manufacturers.

The Oncomine and cBioPortal databases 69, 70 were used to assess the clinical correlations of indicated gene expression levels in breast normal tissues versus (vs.) cancers (Figure 7D-H). Kaplan-Meier Plotter 98, a web-tool with pooled transcriptome analysis of multiple GEO datasets generated using the GEO platforms GPL96, GPL570, and GPL571 which share the same probe sets, was used to analyze the gene expression levels linked to the probability of OS, RFS or PPS in breast cancer patients (Figure 7I-O). For survival analysis generated by Kaplan-Meier Plotter, patients were divided into two cohorts with high and low mean expression of the selected genes as per the best available cut-off values from the lower quartile to the upper quartile of gene expression 99. The indicated survival data were exported to GraphPad Prism software version 10.1.2 (GraphPad, San Diego, CA) for statistical analysis and visualization. TIMER 2.0 100 estimated the correlation of MCTS1 with M2 macrophage or with Tregs infiltration in The Cancer Genome Atlas Breast Invasive Carcinoma (TCGA-BRCA) samples using CIBERSORT-ABS algorithm. Expression levels are indicated as log2 transcripts per million (TPM).

### Statistical analysis

One-way or two-way ANOVA and a two-tailed unpaired Student's *t-*test were used to calculate the statistical significance of pairwise comparisons. Univariate and multivariate Cox regression analyses and log-rank (Mantel-Cox) test were used to analyze the hazard ratio (95% confidence interval) and the statistical significance associated with the indicated genes and clinical attributes. Chi-squared (χ^2^) and two-sided Fisher's exact tests examined the proportion of breast cancer and TNBC patients with the indicated genes/protein compared with normal breast tissue respectively. Pearson's or Spearman's correlation coefficient was used to measure the association between two genes or statistical relevance in the breast cancer cDNA arrays and Oncomine database 69 following the normality test. Spearman's correlation (rho) was used to measure the correlation of MCT-1 and the infiltration of immune cells generated by TIMER2.0 100.

## Supplementary Material

Supplementary methods, figures and tables.

## Abbreviations

Arg-1: arginase-1
BCSCs: breast cancer stem cells
CXCL7: chemokine (C-X-C motif) ligand 7
CXCR2: C-X-C chemokine receptor 2
CTLs: cytotoxic T cells
DEGs: differentially expressed genes
EGFR: epidermal growth factor receptor
EMT: epithelial-to-mesenchymal transition
FLAG-CXCL7: FLAG-tagged CXCL7
GSEA: gene get enrichment analysis
HER2: human epidermal growth factor receptor 2
IFNα: interferon-alpha
IFNγ: interferon-gamma
IL-6: interleukin-6
IL-6R: IL-6 receptor
IL8RB: interleukin-8 receptor beta
i.p.: intraperitoneal
i.v.: intravenous
IVIS: *in vivo* imaging system
LPS: lipopolysaccharide
LN-POS: lymph node metastasis
MSCs: mesenchymal stem cells
MCT-1: multiple copies in T-cell malignancy 1
NK: natural killer
OS: overall survival
PMA: phorbol 12-myristate 13-acetate
PPS: post progression survival
PD-1: programmed cell death protein-1
PD-L1: programmed cell death-1-ligand-1
PR: progesterone receptor
rhCXCL7: recombinant human CXCL7
RFS: recurrence-free survival
Tregs: regulatory T cells
shCXCL7: silencing CXCL7
shIL-6: silencing IL-6
shMCT-1: silencing MCT-1
TNBCs: triple-negative breast cancers
TAMs: tumor-associated macrophages
TILs: tumor-infiltrating lymphocytes
TME: tumor microenvironment
V5-MCT-1: V5-tagged MCT-1
vs.: versus
