# Supplementary material for: Immunotherapeutic IL-6R and targeting the MCT-1/IL-6/CXCL7/PD-L1 circuit prevent relapse and metastasis of triple-negative breast cancer
Source: Theranostics. 2024 Mar 3;14(5):2167–89. doi: 10.7150/thno.92922 (PMC10945351; doi:10.7150/thno.92922)
Supplement: Supplementary file 1 — Supplementary methods, figures and tables. [file thnov14p2167s1.zip › Supplementary Figures (92922i1).pdf]

Supplementary Figures

Immunotherapeutic IL-6R and targeting the MCT-1/IL-6/CXCL7/PD-L1 circuit prevent relapse and metastasis of triple-negative breast cancer

Aushia Tanzih Al Haq[1§], Pao-Pao Yang[1§], Christopher Jin[2], Jou-Ho Shih[2], Hong-Yu Tseng[1], Li-Mei Chen[1], Yen-An Chen[1], Yueh-Shan Weng[1], Lu-Hai Wang[3], Michael P. Snyder[2], Hsin-Ling Hsu[1*]

Figure S1

GSEA leading edge genes: HALLMARK (H) MSigDB
shMCT-1 vs. scramble

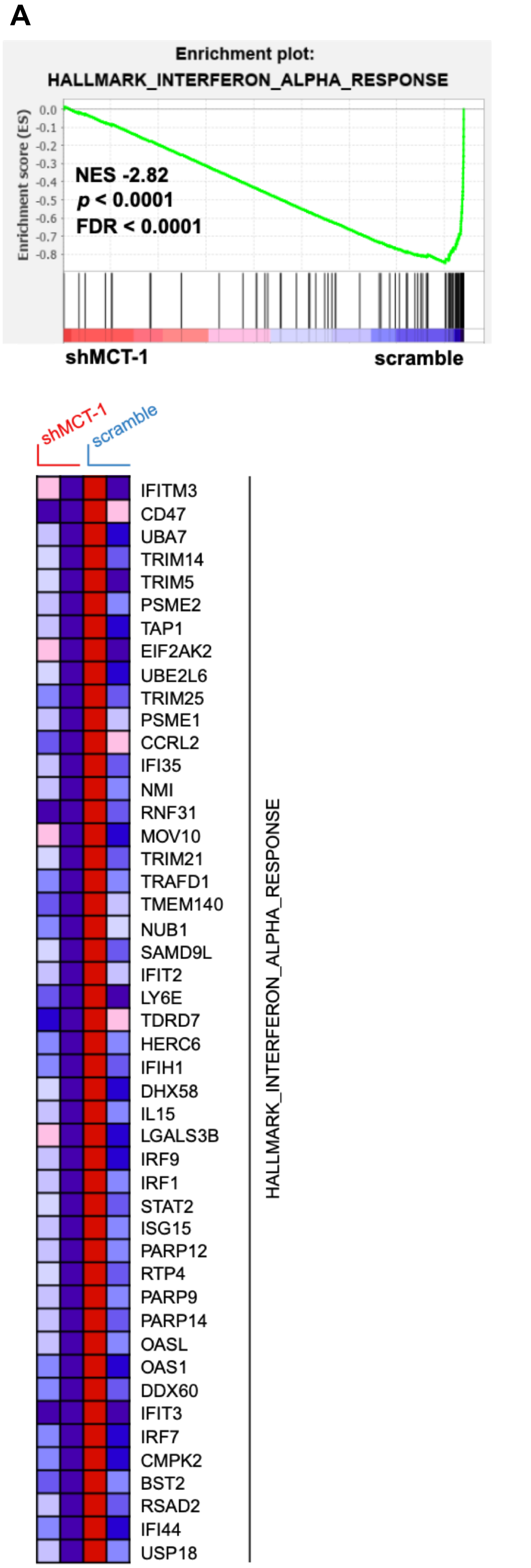

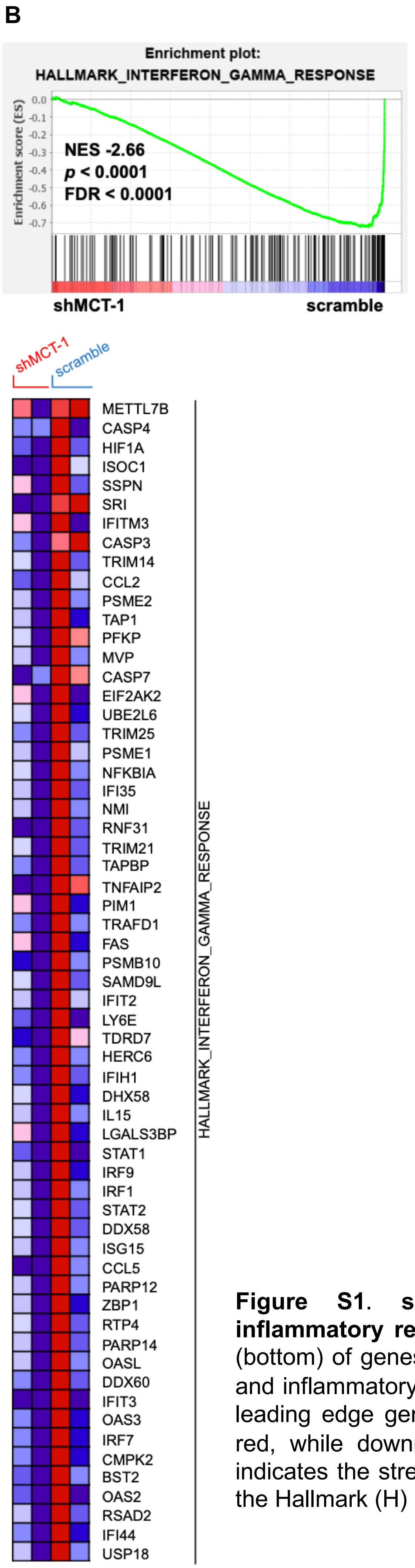

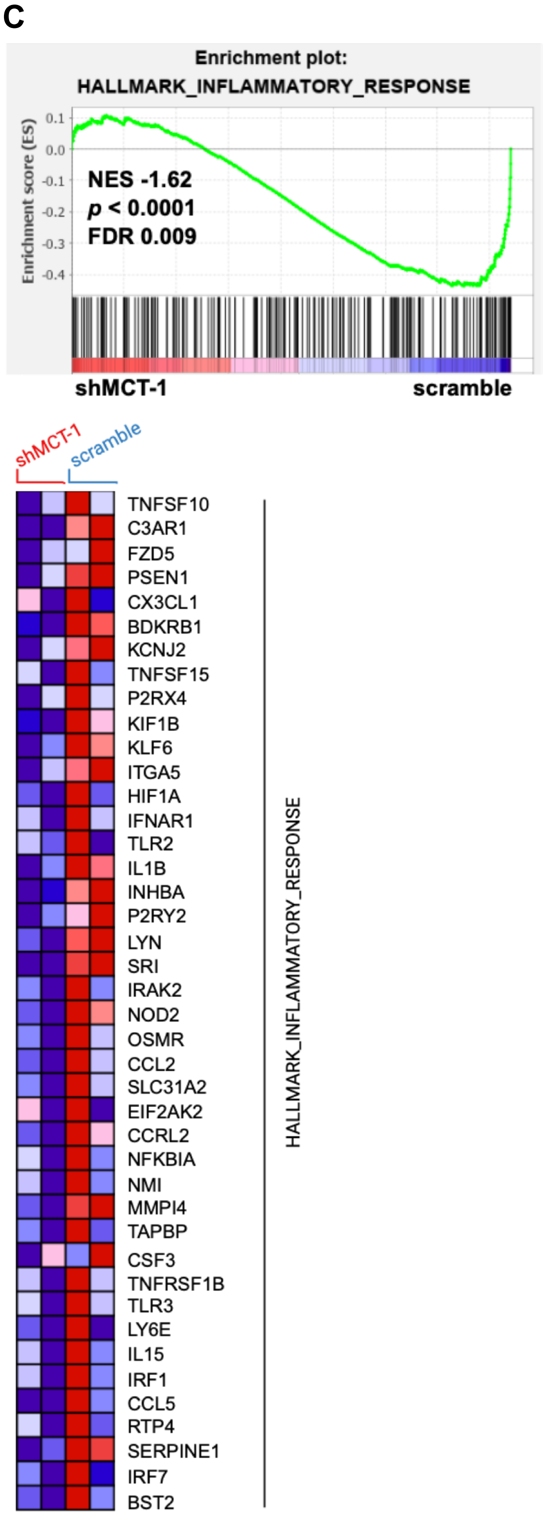

Figure S1. shMCT-1 downregulates interferon and inflammatory responses. Enrichment plot (top) and heatmap (bottom) of genes in interferon-alpha (A), interferon-gamma (B) and inflammatory response (C) gene sets as identified from the leading edge gene list from GSEA. Upregulation is marked in red, while downregulation is marked in blue. Color intensity indicates the strength of regulation. Gene sets are collection of the Hallmark (H) MSigDB.

Figure S2

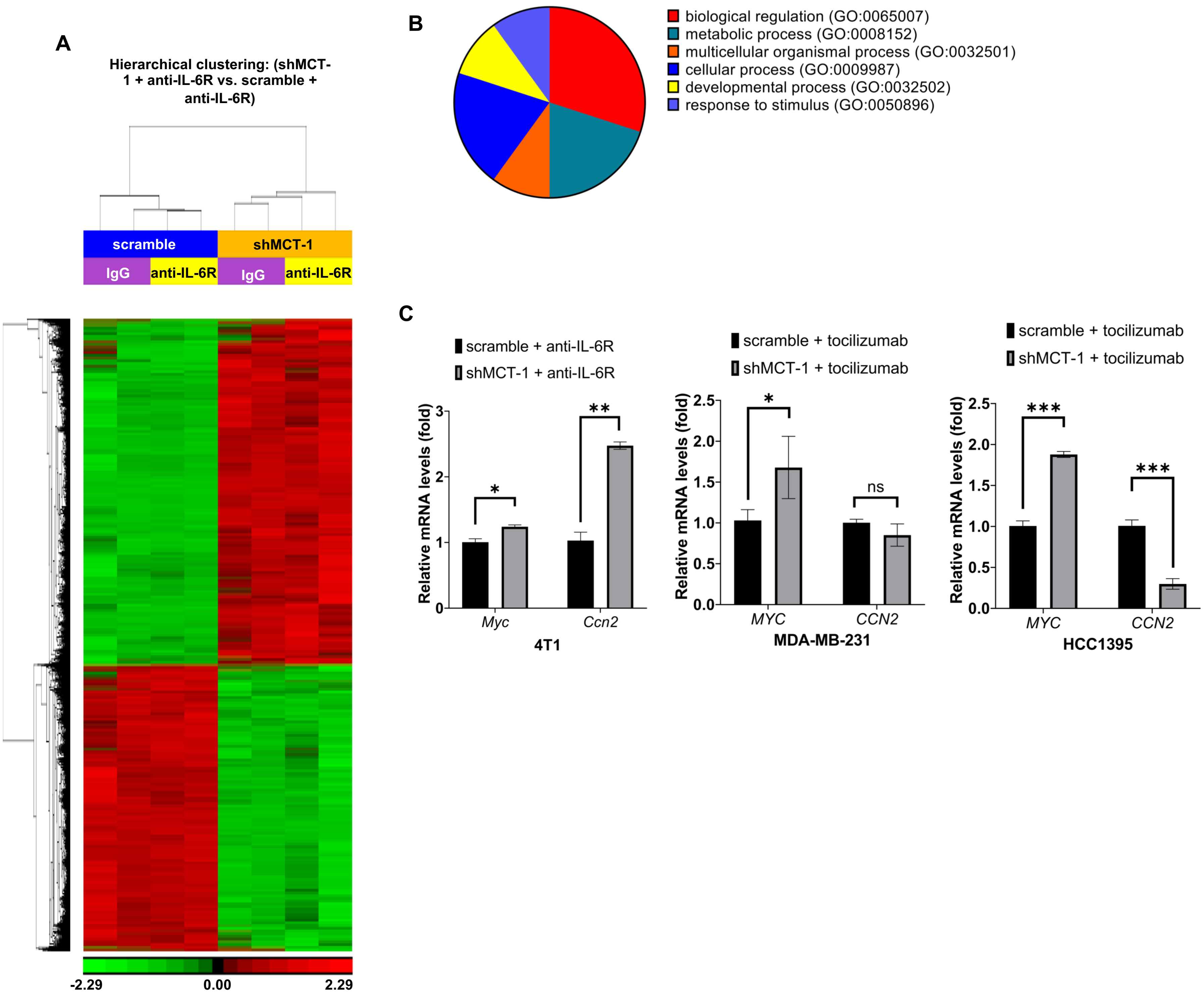

Figure S2. **Common targets between shMCT-1 and anti-IL-6R involve in biological regulation, cellular process and metabolic process.** (A) Hierarchical clustering of DEGs in shMCT-1 vs. scramble within anti-IL-6R treatment (shMCT-1 + anti-IL-6R vs. scramble + anti-IL-6R). Two biologically independent samples for each group were used (red, upregulated; green, downregulated). (B) The pie chart showing Gene Ontology (GO) with Biological Process (BP) subontology classification of common targets between shMCT-1 and anti-IL-6R as characterized using the Protein Analysis Through Evolutionary Relationships (PANTHER) system. GO term and (GO ID) were shown. (C) Quantitative RT-PCR analysis of MYC and CCN2 in anti-mouse IL-6R- or tocilizumab (a humanized anti-IL-6R) treated shMCT-1 as compared to scramble counterpart in murine TNBC 4T1 and human TNBC MDA-MB-231 (IV2-3) and HCC1395 cell lines (n=4). The relative mRNA levels were normalized to the β-actin level and then compared with those in anti-mouse IL-6R- or tocilizumab-treated scramble cells. Statistical significance was determined by a two-tailed unpaired Student's *t*-test and corrected by the Holm-Sidak test. *$p < 0.05$, **$p < 0.01$, ***$p < 0.001$.

Figure S3

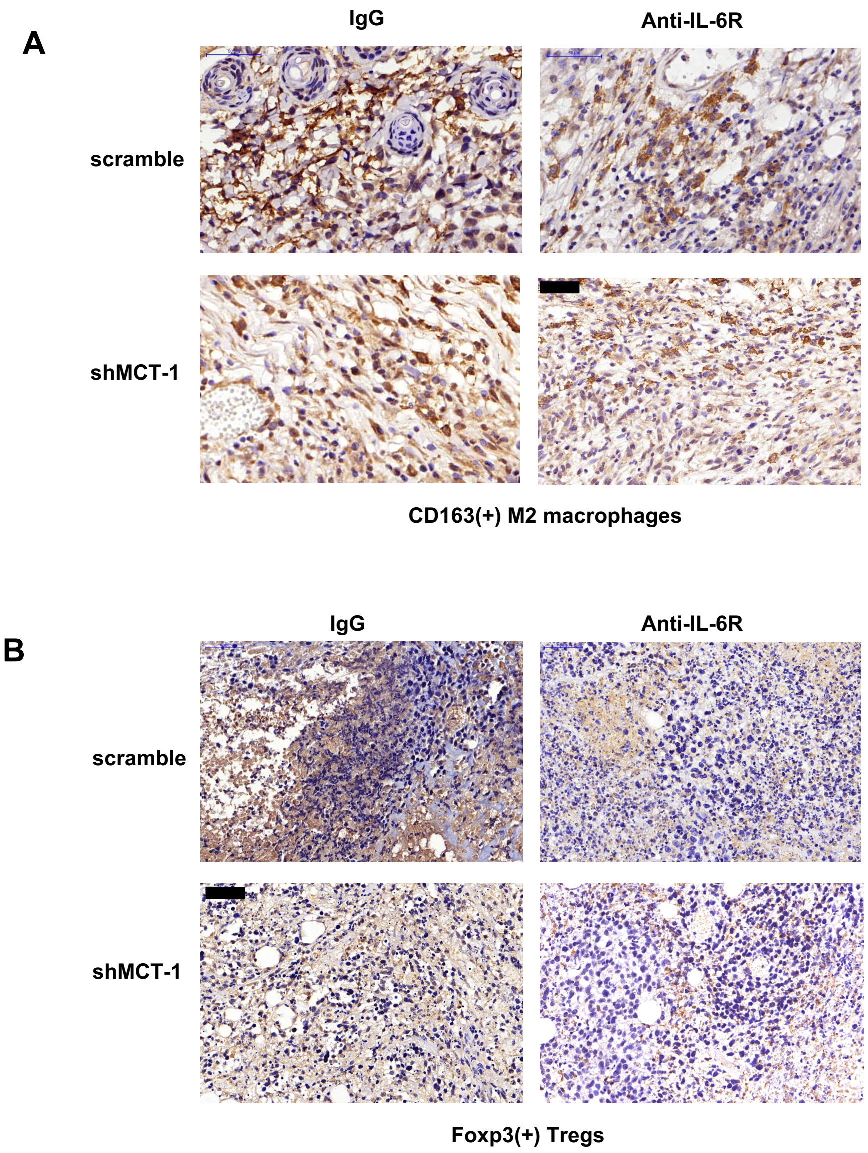

Figure S3. **shMCT-1 improves anti-IL-6R effect against intratumoral M2 macrophages and Tregs.** Immunohistochemistry of CD163(+) M2 macrophages (A) and Foxp3(+) Tregs (B) in the recurrent 4T1 shMCT-1 vs. scramble tumors upon administration of anti-mouse IL-6R Ab (Anti-IL-6R) vs. IgG2b, κ. Scale bar, 50 μm.

Figure S4

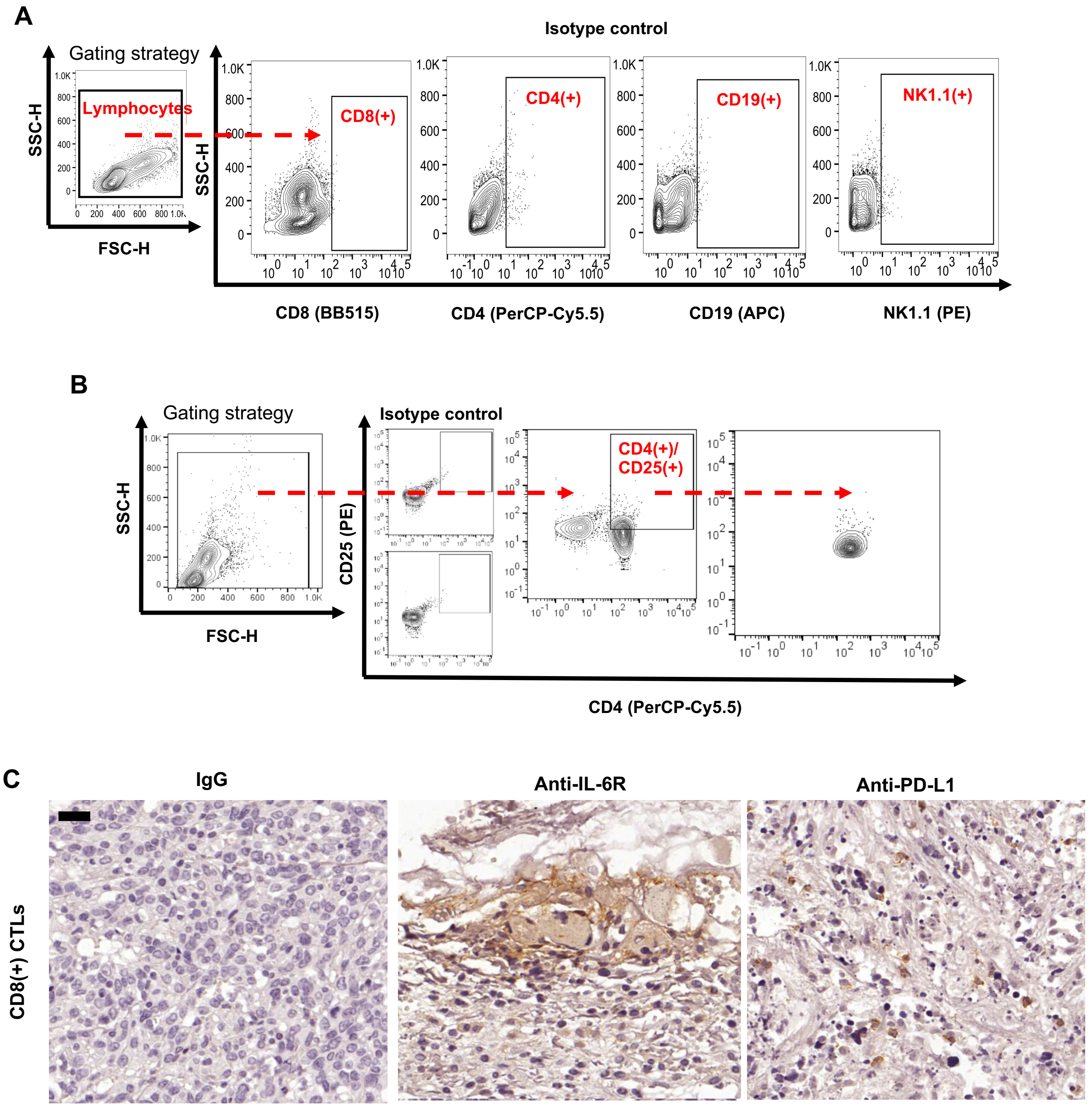

Figure S4. **The gating strategy and immunohistochemistry analysis for defining splenic lymphocytes, Tregs and tumor-infiltrating CD8 T cells.** (A) For flow cytometric analysis of splenic lymphocytes, biotinylated then streptavidin-bound CD8(+), CD4(+), CD19(+) and NK1.1(+) positive fractions were examined for their integrity using side scatter (SSC) vs. forward scatter (FSC) dot plot. Total lymphocytes from the SSC/FSC gate were used to identify CD8(+), CD4(+), CD19(+) and NK1.1(+) subsets based on CD8(+) vs. SSC, CD4(+) vs. SSC, CD19(+) vs. SSC and NK1.1(+) vs. SSC rectangular gating, respectively. Fluorophore-matched isotype control Abs of CD8, CD4, CD19 and NK1.1 were used as negative control for background staining. (B) For flow cytometric analysis of Tregs, the integrity of positive fractions of CD4(+) population was assessed using SSC vs. FSC dot plot (left panel). CD4(+) positive fractions were gated for CD4(+)/CD25(+) cells (right panel) by rectangular gating. Fluorophore-matched isotype control Abs of CD25 and CD4 (middle panel) were used as negative control for background staining. (C) Infiltration of CD8(+) cytotoxic T cells (CTLs) into the 4T1 recurrence breast tumors derived from scramble group with IgG2b, κ, anti-IL-6R or anti-PD-L1 treatment. Representative fields were shown. Scale bar, 50 μm.

Figure S5

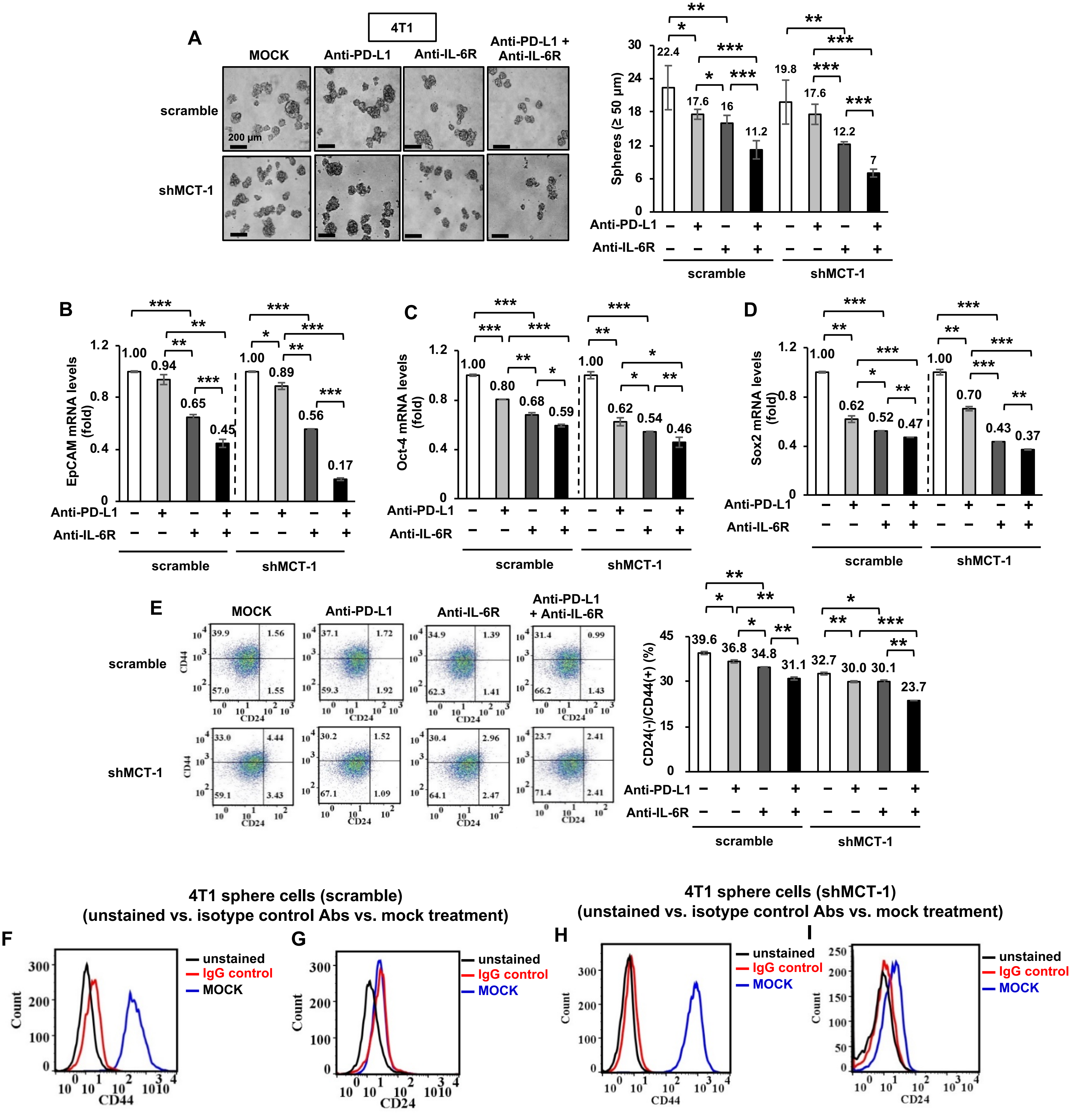

Figure S5. Cancer stemness properties changed by the indicated immunotherapy. (A) The ability of 4T1 cells (scramble vs. shMCT-1) to form mammospheres was measured following anti-mouse IL-6R Ab (Anti-IL-6R) and/or anti-human PD-L1 Ab (atezolizumab) (Anti-PD-L1) treatment for 5 days (n=5). Scale bar, 200 μm. (B-D) EpCAM (B), Oct4 (C) and Sox2 (D) expression levels were analyzed by qRT-PCR (n=5). (E) CD24(-)/CD44(+) subpopulations in the immunotherapy-treated spheres were analyzed by flow cytometry (n=5). (F-I) Flow cytometry analysis of unstained, IgG2b, κ-treated and MOCK-treated 4T1 cells of CD44(+) (F, H) or CD24(+) (G, I) populations in the scramble (F-G) and the shMCT-1 (H-I) conditions. Data are presented as the mean ± SEM. One-way ANOVA with the Tukey-Kramer post hoc test was used to evaluate the statistical significance of pairwise comparisons. *$p < 0.05$, **$p < 0.01$, ***$p < 0.001$.

Figure S6

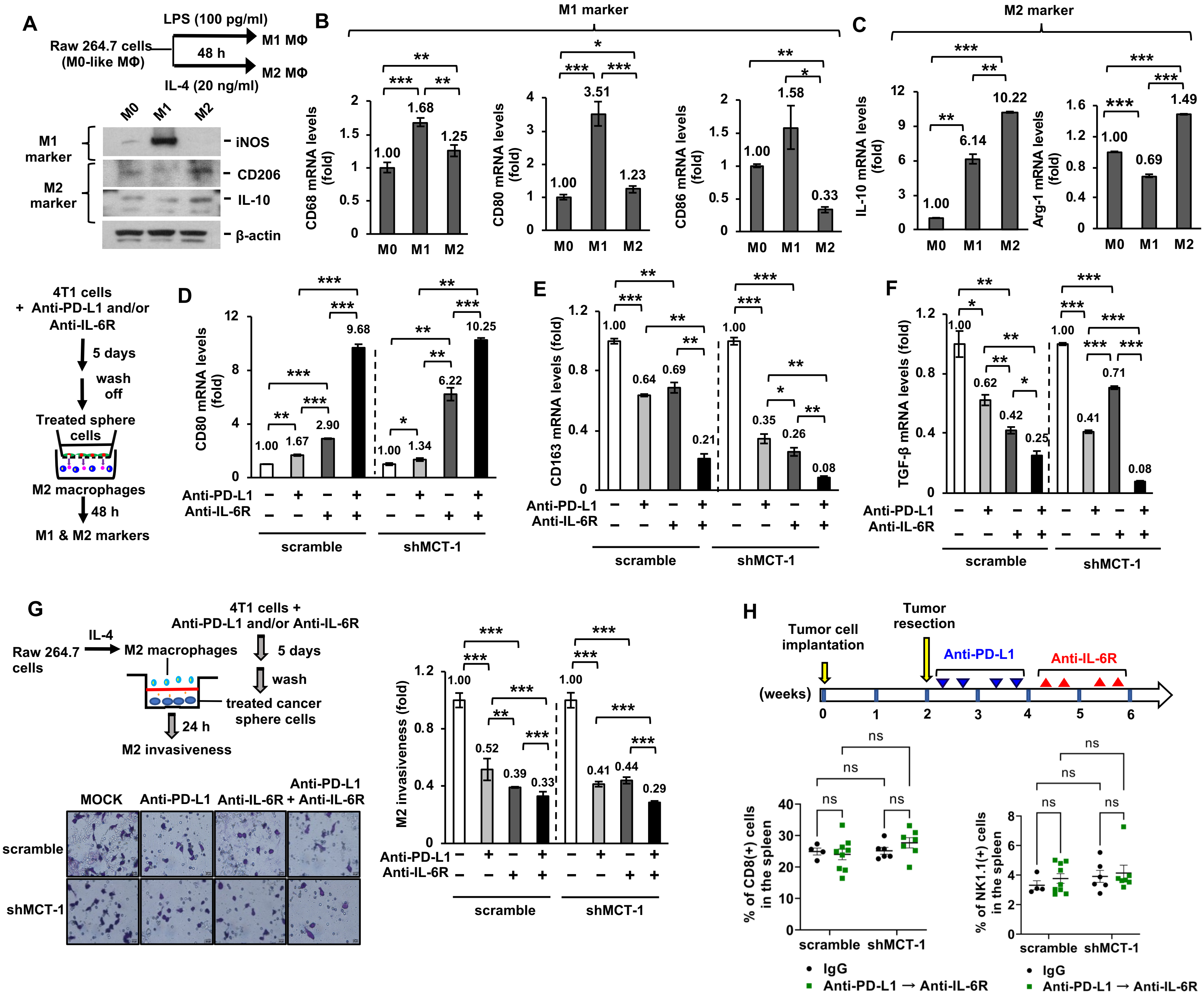

Figure S6. Immunotherapy of BCSCs inhibits M2 macrophage polarity and invasiveness. Murine Raw 264.7 M0-like macrophages were treated with LPS (100 pg/ml) or IL-4 (20 ng/ml) for 48 h to induce M1 and M2 polarization, respectively. (A) Intracellular markers for M1 (iNOS) and M2 (CD206 and IL-10) macrophages were examined in Raw264.7 M0-like macrophages and the polarized M1 and M2 macrophages. (B-C) The mRNA expression levels of M1 (CD68, CD80 and CD86) (B) and M2 (IL-10 and Arg-1) (C) markers were determined by qRT-PCR (n=5). (D-F) Anti-IL-6R and/or anti-PD-L1 immunotherapy-pretreated BCSCs were placed in the upper chamber and used to define the macrophage polarity as cocultured with the Raw 264.7 M2 macrophages in the lower chamber for 48 h. M1 (CD80) (D) and M2 (CD163 and TGF-β) (E-F) markers were determined by qRT-PCR (n=5). (G) The M2 macrophages penetrating through the upper chamber of an invasion transwell were evaluated (n=5) after coculture with the immunotherapy-treated BCSCs in the lower chamber for 24 h. (H) Splenic CD8(+) CTLs and NK cells were detected in the 4T1 xenograft mice after the sequential immunotherapy (Anti-PD-L1 → Anti-IL-6R). Data are presented as the means ± SEM. One-way (B-G) and two-way (H) ANOVA with the Tukey-Kramer post hoc test were used to calculate statistical significance. ns: not significant, *$p < 0.05$, **$p < 0.01$, ***$p < 0.001$.

Figure S7

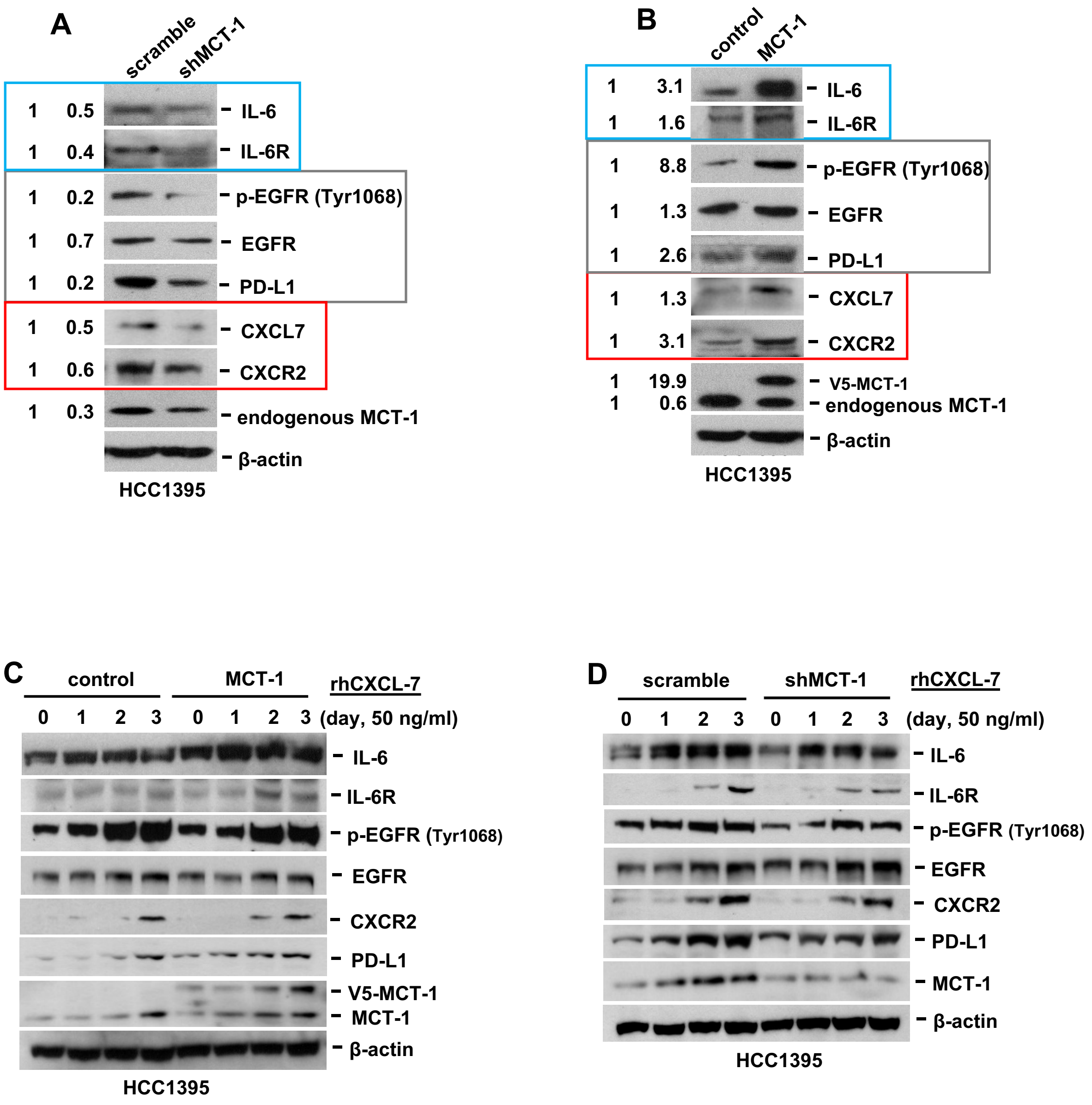

Figure S7. MCT-1/CXCL7 promotes IL-6/EGFR/PD-L1 in TNBC cells. The indicated proteins were analyzed in HCC1395 cells with or without MCT-1 knockdown (scramble vs. shMCT-1) (A) and MCT-1 overexpression (control vs. MCT-1). Following rhCXCL7 treatment (0~3 days), the indicated proteins were assayed in HCC1395 with MCT-1 overexpression (C) or depletion (D). The relative protein level was normalized to the internal β-actin level and then compared with that in the control or scramble cells (A-D).

Figure S8

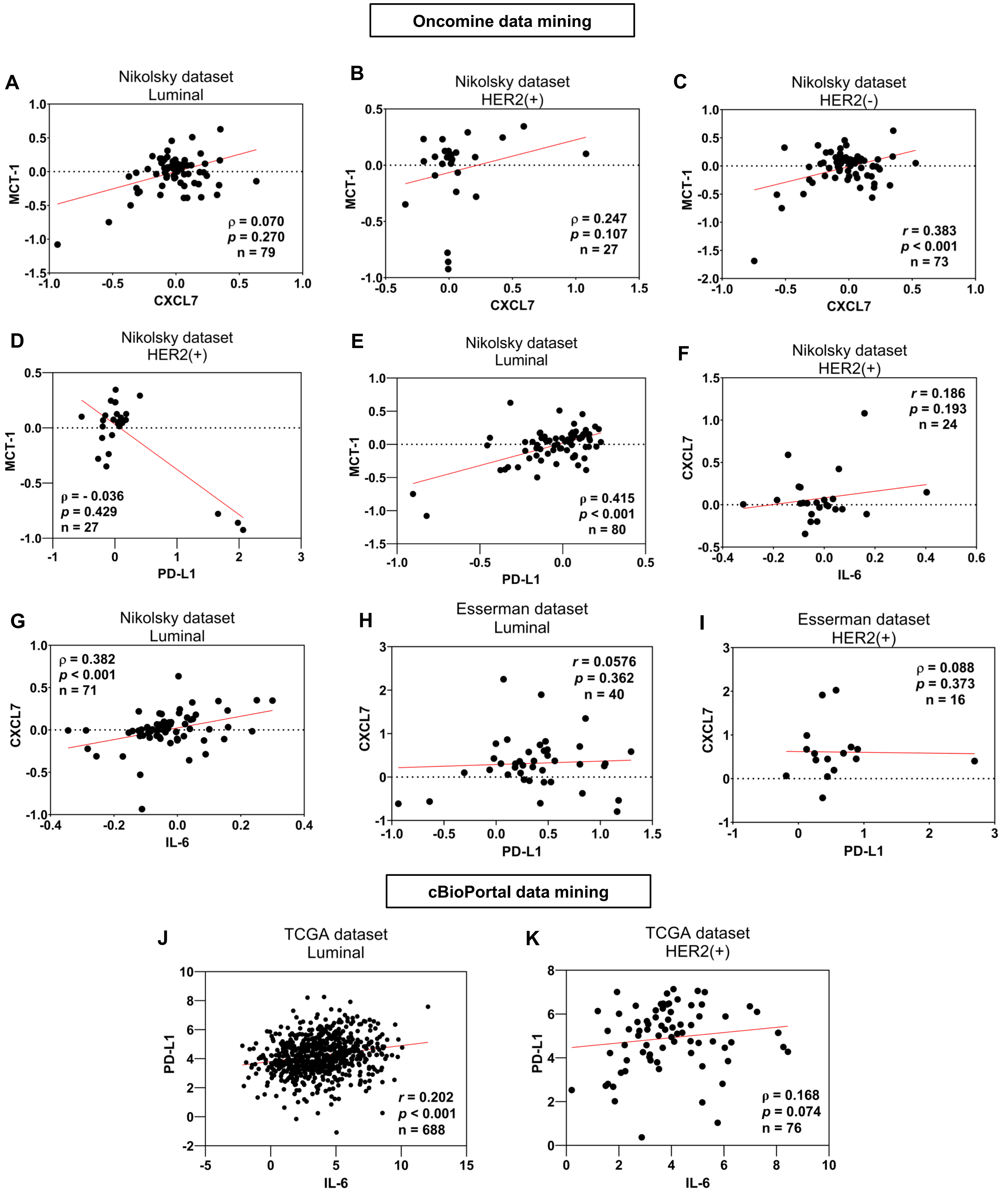

Figure S8. **ER and HER2 status determine the clinical correlation of MCT-1/IL-6/CXCL7/PD-L1 circuit.** Scatter plots of gene expression of MCT-1 vs. CXCL7 (A-C), MCT-1 vs. PD-L1 (D-E), CXCL7 vs. IL-6 (F-G), CXCL7 vs. PD-L1 (H-I) and PD-L1 vs. IL-6 (J-K) in the indicated breast cancer molecular subtypes. Significance was determined by one-tailed Pearson's (denoted by *r*) or Spearman's (denoted by ρ) correlation test. Regression line was shown in red solid line. mRNA expression is reported as fold change (A-I) or RSEM (batch normalized from Illumina HiSeq_RNASeqV2) (log2). Datasets were obtained from Oncomine (A-I) and cBioPortal (J-K) databases then analyzed and visualized using GraphPad Prism 10.1.2.
