# Supplementary material for: Immunotherapeutic IL-6R and targeting the MCT-1/IL-6/CXCL7/PD-L1 circuit prevent relapse and metastasis of triple-negative breast cancer
Source: Theranostics. 2024 Mar 3;14(5):2167–89. doi: 10.7150/thno.92922 (PMC10945351; doi:10.7150/thno.92922)
Supplement: Supplementary file 1 — Supplementary methods, figures and tables. [file thnov14p2167s1.zip › Supplementary Methods.pdf]

Immunotherapeutic IL-6R and targeting the MCT-1/IL-6/CXCL7/PD-L1 circuit prevent relapse and metastasis of triple-negative breast cancer

Aushia Tanzih Al Haq[1§], Pao-Pao Yang[1§], Christopher Jin[2], Jou-Ho Shih[2], Hong-Yu Tseng[1], Li-Mei Chen[1], Yen-An Chen[1], Yueh-Shan Weng[1], Lu-Hai Wang[3], Michael P. Snyder[2], Hsin-Ling Hsu[1]*

Supplementary Materials

The supplementary materials include:

Supplementary Tables (as separate .xls files)

Table S1. DEGs of shMCT-1 vs. scramble

Table S2. GSEA MSigDB Hallmark (H) gene sets: shMCT-1 vs. scramble

Table S3. Annotated transcripts and pairwise DEGs of shMCT-1 vs. scramble (anti-IL-6R vs. IgG)

(A) Sample annotation, processed, and normalized data

(B) scramble + anti-IL-6R vs. scramble + IgG

(C) shMCT-1 + anti-IL-6R vs. shMCT-1 + IgG

(D) shMCT-1 + anti-IL-6R vs. scramble + anti-IL-6R

Table S4. DEGs that overlapped between scramble + anti-IL-6R vs. scramble + IgG, shMCT-1 + anti-IL-6R vs. shMCT-1 + IgG, shMCT-1 + anti-IL-6R vs. scramble + anti-IL-6R pairwise comparisons

Table S5. GSEA MSigDB Hallmark (H) gene sets: shMCT-1 vs. scramble (anti-IL-6R vs. IgG)

(A) scramble + anti-IL-6R vs. scramble + IgG

(B) shMCT-1 + anti-IL-6R vs. shMCT-1 + IgG

(C) shMCT-1 + anti-IL-6R vs. scramble + anti-IL-6R

Table S6. Primer sequences for specific amplicons

Supplementary Figures (as separate .PDF file)

Figure S1. shMCT-1 downregulates interferon and inflammatory responses.

Figure S2. Common targets between shMCT-1 and anti-IL-6R involve in biological regulation, cellular process and metabolic process.

Figure S3. shMCT-1 improves anti-IL-6R effect against intratumoral M2 macrophages and Tregs.

Figure S4. The gating strategy and immunohistochemistry analysis for defining splenic lymphocytes, Tregs and tumor-infiltrating CD8 T cells.

Figure S5. Cancer stemness properties changed by the indicated immunotherapy.

Figure S6. Immunotherapy of BCSCs inhibits M2 macrophage polarity and invasiveness.

Figure S7. MCT-1/CXCL7 promotes IL-6/EGFR/PD-L1 in TNBC cells.

Figure S8. ER and HER2 status determine the clinical correlation of MCT-1/IL-6/CXCL7/PD-L1 circuit.

Supplementary methods and references

Supplementary Methods

Cell culture

TNBC cell lines (4T1, HCC1395 and MDA-MB-231 (IV2-3)) and murine M0 macrophage-like RAW264.7 cells were cultured in RPMI-1640 medium (Life Technologies, Grand Island, NY) supplemented with 10% fetal bovine serum (FBS) (Life Technologies), 100 units/ml penicillin (HyClone, South Logan, UT), 100 μg/ml streptomycin (HyClone) and 2 mM L-glutamine (HyClone). Cells were cultured in an incubator with 5% CO_2 at 37°C.

Western blot analysis, antibodies (Abs) and reagents

Cell lysates were subjected to western blot analysis as previously described [1]. An anti-human IL-6R Ab (for Western blotting) was purchased from PeproTech (Rocky Hill, NJ). Abs against phospho-EGFR (Tyr1068), EGFR, phospho-Src (Tyr416), Src, phospho-Stat3 (Ser727), phospho-Stat3 (Tyr705), Stat3, IL-6, PD-L1, iNOS, AKT and phospho-AKT (Ser473) were obtained from Cell Signaling Technology, Inc. (Danvers, MA). Abs against ZEB1 and Twist1 were bought from Santa Cruz Biotechnology, Inc. (Dallas, Texas). Abs against endogenous MCT-1 (N1C3), BIM1, Nanog, SOX2, EpCAM and β-actin were acquired from GeneTex (Irvine, CA). Abs against IL-10, CXCL7 and CXCR2 were ordered from Elabscience (Houston, TX). Ab against CCN2 was purchased from ABclonal (Woburn, MA). Recombinant human CXCL7 (R&D Systems, Minneapolis, MN), IL-6 (PeproTech, Rocky Hill, NJ), anti-PD-L1 Ab (atezolizumab), gefitinib (IRESSA) (ACE Biolabs, Taoyuan, Taiwan), anti-mouse IL-6R Ab (Bio X Cell, Lebanon, NH).

Plasmid construction and transfection

Full-length MCT-1 cDNA purified from normal breast epithelial cells (MCF-10A) was cloned into the pLXSN retroviral vector as described previously [2]. The viral supernatants were incubated with highly invasive MDA-MB-231 (IV2-3) and HCC1395 TNBC cells for 24 h, and the infected cells were then refed with fresh medium and cultured for another 24 h. Transfectants were selected with 400 μg/ml neomycin (G418) (Millipore, Darmstadt, Germany) for 2 weeks. Stable clones were confirmed by Western blot analysis by an anti-V5 Ab (Invitrogen, Carlsbad, CA) as described previously [3].

The pcDNA3.1 vector (Invitrogen) and pcDNA3.1-CXCL7-FLAG (GenScript, Piscataway, NJ) were further transfected into the MDA-MB-231 (IV2-3) cells with different levels of MCT-1 expression using Lipofectamine 3000 (Invitrogen), selected with neomycin G418 (200 μg/ml)-containing medium for 2 weeks and confirmed by anti-FLAG Ab (Sigma-Aldrich) immunoblotting.

Cancer stemness

A single-cell suspension was cultured in a 6-well ($4x10^4$ cells/well) ultralow-attachment plate (Corning, Corning, NY) in serum-free Dulbecco’s modified Eagle’s medium/F12 (Life Technologies) supplemented with 1% L-glutamine (HyClone), 1% penicillin/streptomycin (HyClone), 2% B27

(Invitrogen), 20 ng/ml EGF (Sigma-Aldrich) and 20 ng/ml FGFb (PeproTech). An anti-human IL-6R mAb (tocilizumab) (CHUGAI Pharmaceuticals) (200 μg/ml), anti-mouse IL-6R Ab (Bio X Cell) (20 μg/ml) and/or anti-PD-L1 Ab (atezolizumab) (50 μM) (Selleckchem, Houston, TX) were used to treat 4T1 cancer cells for 5~7 days. Mammospheroids were observed at a magnification of 200× using a Nikon DIAPHOT300 microscope (Nikon, Tokyo, Japan). Immunotherapy-treated 4T1 sphere cells ($1x10^5$) were stained with Alexa Fluor 700-conjugated anti-CD24 (BD Biosciences, Franklin Lakes, NJ) and BB515-conjugated anti-CD44 (BD Biosciences) at 1:50 dilutions for 1 h at 4°C. Alexa Fluor 700-conjugated and BB515-conjugated rat IgG2b, κ were used as isotype control Abs (BD Biosciences) for 1 h at 4°C, washed and then resuspended in 500 μl phosphate-buffered saline (PBS) (137 mM NaCl, 2.7 mM KCl, 10 mM Na_2HPO_4, 1.8 mM KH_2PO_4, pH 7.4). Anti-CD24-Alexa Fluor 700 and anti-CD44-BB515 were excited at 633 nm and 488 nm, and their emission signals were determined by the FL-4 PMT (653-669 nm bandpass filter) and FL1 PMT (515-545 nm bandpass filter), respectively. CD44(+)/CD24(-) BCSC subpopulations were analyzed with a BD FACSCalibur flow cytometer and Cell Quest software (BD Biosciences).

Cancer stemness markers were examined in immunotherapy-treated mammospheres by qRT-PCR as described previously [4]. The primers (Table S3) for specific amplicons were synthesized (MDBio, Taipei, Taiwan) and designed according to the NCBI Probe database.

M2 macrophage polarity and invasion

We analyzed macrophage activity in response to the immunotherapy treated BCSCs. M2 macrophages were differentiated from Raw264.7 M0 macrophages after treatment with IL-4 (20 ng/ml) (PeproTech) for 48 h. 4T1 cancer cells were cultured in a low-attachment plate (Corning, Corning, NY) and treated with humanized anti-PD-L1 (atezolizumab) Ab (50 μM) (Selleckchem) and/or anti-mouse IL-6R Ab (20 μg/ml) (Bio X Cell) for 5 days. The immunotherapy-pretreated 4T1 cells ($4x10^5$) were rinsed and seeded in the upper chamber of Falcon® Cell Culture Inserts (Corning) and incubated for 48 h with the derived M2 macrophages (1×10^6) placed in the bottom chamber. A control experiment was conducted with M2 macrophages when cocultured with untreated BCSCs.

Macrophage polarity was evaluated by quantitative reverse transcription-polymerase chain reaction (qRT-PCR) analysis of indicated markers. Cellular RNAs of the polarized macrophages were isolated in a TRIzol solution (Thermo Fisher Scientific), treated with DNase I and transcribed into cDNA by SuperScript™ II reverse transcriptase (Thermo Fisher Scientific) according to manufacturer's instructions. Quantitative RT-PCR was conducted using SYBR Green Master Mix, a cDNA template (100 ng) and the indicated primers (online supplemental figure S6). The qRT-PCR reaction was performed at 95°C for 15 min, followed by 40 cycles at 95°C for 15 seconds and 60°C for 1 min. The β-actin expression level was used as an internal control. Relative mRNA levels were calculated by the formula: ΔΔCT=ΔCt test sample-ΔCt control sample. Fold changes in gene expression were calculated using the $2^{-\Delta\Delta CT}$ method.

The ability of M2 macrophages penetrating through the upper chamber of an invasion transwell was evaluated after coculture with the immunotherapy treated BCSCs in the lower chamber for 24 h.

Quantitative RT-PCR analysis of lung tissues

RNA*later*-stored (Invitrogen, Carlsbad, CA) murine metastatic lung tissues (~20 mg) were homogenized with TRIzol Reagent (Invitrogen) using TissueLyser II (Qiagen GmbH, Hilden, Germany) at 30 Hz for 2 x 3 minutes following manufacturer's protocol. To synthesize cDNA, 2 µg of total RNA was used for oligo dT primed reverse-transcription using FIREScript RT cDNA synthesis kit (Solis BioDyne, Tartu, Estonia). cDNA samples (100 ng) were subjected to qRT-PCR with HOT FIREPol SolisGreen qPCR Mix (Solis BioDyne) using a ViiA 7 Real-time PCR System (Applied Biosystems, Carlsbad, CA). *Ppia* was used as an internal control. Results were analyzed using comparative Ct methods ($2^{-\Delta\Delta Ct}$). ΔΔCT = [(Ct target gene − Ct internal control) of immunotherapy-treated mice − (Ct target gene − Ct internal control) of IgG-treated mice]. Primers used in the qRT-PCR are in Table S3.

Gene knockdown

pGeneClip MCTS1 shRNAs (SA Biosciences Corp, Frederick, MD) (#2: 5'-AGTCCGATGCCATGAACATAT-3', #3: TGCTGCAGTAGATACCATTGT for targeting the MCT-1 gene in human MDA-MB-231 (IV2-3) and HCC1395 cells, and 5'-TCCTTACAGTAAATGGAGAAT-3' for targeting the MCT-1 gene in murine 4T1 cells) and scramble shRNA (5'-GGAATCTCATTCGATGCATAC-3') (SA Biosciences Corp) were stably transfected into indicated cell lines using jetPEI transfection reagent (Polyplus-transfection, New York, NY) following the manufacturer's protocol. Transfectants were selected in medium containing 0.5 µg/ml puromycin (Millipore, Darmstadt, Germany) for 2 weeks.

To target the IL-6 gene, a pool of three target-specific lentiviral vector plasmids (Santa Cruz, Dallas, TX), each encoding 19- to 25-nucleotide shRNAs, was used. The IL-6-specific shRNA and scramble viral vectors (5 µg) were individually transfected into 293T cells. The viral supernatants were collected, infected cancer cells and selected stable lines with 0.5 µg/ml puromycin-containing medium for 2 weeks.

A CXCL7-specific shRNA nucleotide (5'-TGATCGGGAAAGGAACCCATT-3') was inserted into the pLKO.1 vector (RNAi Core Academia Sinica, Taipei). The CXCL7-specific shRNA-containing and scramble viral vectors (5 µg) were individually transduced into 293T cells. The viral supernatants were transfected into MDA-MB-231 (IV2-3) cells and then selected with puromycin (6 µg/ml)-containing medium for 2 weeks.

Plasma IL-6 levels

Blood samples were collected from immunotherapy-treated mice and placed into microtainer tubes with K_2EDTA (BD, Franklin Lakes, NJ). After centrifugation (1000 x g) for 10 min at 4°C, the

plasma was kept at -80°C until analysis. IL-6 levels were quantified by an assay performed with the MAX Deluxe Set Mouse IL-6 (BioLegend, San Diego, CA). Plates were coated with mouse IL-6 ELISA MAX™ capture Ab, incubated overnight at 4 °C, washed with 0.05% Tween-20/PBS and incubated with the assay buffer for 1 h. The plates were incubated with diluted standards or 100-µl plasma samples for 2 h, followed by reactions with a diluted detection Ab for 1 h, Streptavidin-horseradish peroxidase (HRP) for 30 min and a substrate solution in the dark for 20 min. Results were measured on a Tecan Infinite M200 Pro microplate reader (Tecan, Männedorf, Switzerland) and analyzed with Tecan's i-control. 1.2 software.

Assessment of splenic and tumor-infiltrating lymphocytes

Murine spleens were harvested and washed twice in PBS, gently minced in ice-cold RPMI-1640 medium (2% FBS), and then mashed using the plunger of a 1-ml syringe.

Macrophages and Tregs were isolated from breast tumors and lung nodules as previously described [5, 6]. Briefly, tumors and lung tissues were minced and enzymatically digested with a cocktail containing 1 mg/ml collagenase D (Sigma-Aldrich), 0.25 mg/ml DNase I (Sigma-Aldrich), and a 0.25% (v/v) trypsin-EDTA solution (HyClone) in serum-free RPMI-1640 medium. The cell suspension was passed through a 70-µm nylon cell strainer to remove cell clumps, lysed red blood cells with the buffer (155 mM NH_4Cl, 12 mM $NaHCO_3$ and 0.1 mM EDTA) (Alfa Aesar, Ward Hill, MA) at 4^0C for 4 min and then fixed with 4% (v/v) paraformaldehyde at room temperature for 30 min.

Immunophenotyping of splenic and tumor-infiltrating lymphocytes was performed. All the test Abs for characterizing T cells and NK cells were from BD Biosciences (San Jose, CA). Lymphocytes ($1x10^6$) were isolated by reacting with anti-mouse CD16/CD32 (1:2,000) in staining buffer (0.1% bovine serum albumin (BSA) in PBS) to block nonspecific binding. The isolated lymphocytes were stained with cocktails of biotinylated mouse anti-mouse NK-1.1, rat anti-mouse CD19, rat anti-mouse CD4 and rat anti-mouse CD8α at a 1:100 dilution and then purified by positive selection using Streptavidin Particles Plus. The purified lymphocytes were stained with PE-conjugated mouse anti-mouse NK-1.1 (1:25), APC-conjugated rat anti-mouse CD19 (1:50), PerCP-Cy5.5-conjugated Rat anti-mouse CD4 (1:50) and BB515-conjugated rat anti-mouse CD8α (1:100) Abs. Tregs were positively selected with anti-mouse CD4 magnetic particles and then stained with PerCP-Cy5.5-conjugated rat anti-mouse CD4 (1:50) and PE-conjugated rat anti-mouse CD25 (1:25) Abs. BB515-conjugated rat anti-mouse CD8α, PE-conjugated mouse anti-mouse NK-1.1, PE-conjugated rat anti-mouse CD25 and PerCP-Cy5.5-conjugated Rat anti-mouse CD4 were excited at 488 nm, and the emission was detected in the FL1 channel (515-545 nm) with a 530/30 bandpass filter, FL2 channel (564-606 nm) with a 585/42 bandpass filter and FL3 channel with a 670 nm long-pass filter, respectively.

All macrophage sorting reagents were from BioLegend (San Diego, CA). Macrophages were positively selected with anti-CD11b magnetic particles and then stained with BB515-conjugated rat anti-CD11b (1:50), BB700-conjugated rat anti-mouse F4/80 (1:50), PE-conjugated hamster anti-

mouse CD80 (1:25) and Alexa Fluor 647-conjugated anti-mouse CD206 (1:50) Abs. BB515-conjugated rat anti-CD11b, PE-conjugated hamster anti-mouse CD80 and BB700-conjugated rat anti-mouse F4/80 were excited at 488 nm, and the emission was detected in the FL1 channel (515-545 nm) with a 530/30 bandpass filter, FL2 channel (564-606 nm) with a 585/42 bandpass filter and FL3 channel with a 670 nm long-pass filter, respectively. Alexa Fluor 647-conjugated anti-mouse CD206 was excited at 633 nm, and the emission was determined in the FL4 channel (653-669 nm) with a 661/16 bandpass filter.

BD Biosciences was the source of all the isotype control Abs including PE-conjugated hamster IgG2a, κ; BB515-conjugated rat IgG2b, κ; BB700-conjugated rat IgG2a, κ; APC-conjugated rat IgG2a, κ; and PerCP-Cy5.5-conjugated Rat IgG2a, κ. A BD FACSCalibur flow cytometer and FlowJo v10 (BD Biosciences) were used to examine the results.

Immunocytochemistry study

4T1 cells ($1x10^5$) were cultured on a 12-well plate (FALCON, Durham, USA) containing sterile with a micro cover glass inserts (18 mm) (Matsunami GLASS IND., LTD., Osaka, Japan). After 24 h incubation, the cells were pretreated with anti-mouse IL-6R mAb (50 ng/ml) (BE 0047, Bio X Cell) for 24 h, fixed in 4% paraformaldehyde for 10 min, washed twice in PBS and permeabilized in 0.5% Triton X-100/PBS for 5 min and then incubated with blocking buffer (5% BSA/PBS) for 1 h. Afterward, the cells on coverslips were incubated overnight at 4 °C with primary rabbit PD-L1 Ab (#13684) (1:1000) (Cell Signaling Technology) diluted in 5% BSA/PBS, washed thrice with 0.1% Tween 20/PBS (PBS-T) for 5 min, and incubated with the secondary Ab Alexa Fluor 594 goat anti-rabbit IgG (A11037) (1:1000) (Invitrogen) diluted in 5% BSA/PBS for 1 h and washed with PBS-T (3 × 5 min). The coverslips were mounted on slides with Immu-Mount^{TM} medium (Thermo Fisher Scientific Inc.). Images were visualized with a Leica TCS SP5 confocal microscope (Leica, Germany) at × 63 magnifications and analyzed using ImageJ software (version 1.2) (NIH, Bethesda, MD).

Immunohistochemical (IHC) study

Tumors or the normal tissues were fixed in 10% formalin (Sigma-Aldrich), embedded in paraffin, and sliced into 4-μm-thick sections. The paraffin sections were deparaffinized and rehydrated by incubations in xylene (2 × 12 min), 100% ethanol (2 × 10 min), 95% ethanol (2 × 10 min), 80% ethanol (2 × 5 min) and 70% ethanol (2 × 5 min). The samples were washed in distilled water for 5 min, treated with 3% hydrogen peroxide (H_2O_2) for 12 min to block endogenous peroxidase activity and then washed with PBS (3 x 5 min). Antigen retrieval was achieved by heating in sodium citrate-EDTA buffer (10 mM Tri-sodium citrate, 1 mM EDTA, 0.05% Tween 20, pH 6.0) for 16 min in a microwave oven (640 Watt); the sections were then cooled with tap water for 20 min and rinsed with distilled water (4 x 5 min) and PBS for 5 min.

To identify immune cell infiltration into tumor, CD163 is used as a marker for M2 macrophages, whereas Foxp3 is used as a specific marker of mouse Tregs [7]. IHC staining was performed using a

DAKO EnVision detection kit (Agilent Technologies, Santa Clara, CA). Primary Abs against CD163 (GTX35247) (1:600) (GeneTex Inc., Irvine, CA), MCT-1 (GTX117793) (1:200) (GeneTex Inc.) and Foxp3 (GTX107737) (1:100) (GeneTex Inc.) and CD8α (98941) (Cell Signaling Technology Inc.) were incubated with the specimens for 30 min, washed in PBS for 15 min and incubated with a labeled polymer-HRP-conjugated secondary Ab (DAKO EnVision System) (Agilent Technologies, Inc., Santa Clara, USA) for 10 min. The samples were visualized using 3,3'-diaminobenzidine substrates (DAKO EnVision System), counterstained with Mayer’s hematoxylin (Sigma-Aldrich), dehydrated with ethanol (70%, 3 min; 80%, 3 min; 95%, 3 min; and 100%, 5 min), cleared with xylene (5 min) and cover-slipped (Automat-star 24 x 50 mm) with the mounting medium Histokitt solution (Glaswarenfabrik Karl Hecht GmbH & CO KG., Germany).

To analyze lymphangiogenesis in lymph node-metastasized 4T1 tumors, paraffin-embedded tissues were sectioned, deparaffinized and rehydrated as previously described [8]. Briefly, tissue sections were heated in a microwave (640 Watt) in 10 mM sodium citrate containing 0.05% Tween 20, pH 6.0, for 15 min and then rinsed 3 times in PBS. Endogenous peroxidase activity was quenched with 3% H_2O_2 in PBS for 10 min, and then the sections were blocked in 3% BSA in 0.1% Tween 20/PBS. The specimens were incubated with an anti-LYVE Ab (ab33682) (1:100) (Abcam, Cambridge, MA) overnight at 4°C and counterstained with hematoxylin. The immunoreactive signal was developed with a mouse/rabbit polydetector HRP/DAB detection system (BioSB, Santa Barbara, CA).

IHC images were observed under a Nikon Optiphot-2 Upright Microscope using a 40× objective lens and analyzed by the automatic digital slide scanner system Pannoramic MIDI II (3DHISTECH, Ltd., Budapest, Hungary). ImageJ software (version 1.2) was used to conduct color deconvolution as described before [9], adjust the image threshold and analyze the signal intensity. Results were evaluated for at least 6 randomly chosen fields per sample.

To perform immunofluorescence study, the tumor sections were pre-incubated with blocking buffer (5 % BSA diluted in permeabilization solution of 0.2 % gelatin/0.25% Triton/PBS) for 1 h, incubated overnight at 4 °C with a rabbit anti-CD163 antibody (Elabscience, Cat # E-AB-70306), washed by PBS twice and PBS/gelatin/Triton 0.25% for 10 min each and incubated with Alexa Fluor 488 goat anti-rabbit IgG (Invitrogen #A11034, ThermoFisher, Waltham, MA, USA) diluted with blocking buffer (1/200) for 1 h. The reactive samples were rinsed with PBS (3x 10 min), 10 mM $CuSO_4$/50 mM NH_4Cl_4 solution for 10 min and distilled water. Immunofluorescent images were observed under a multispectral Leica TCS SP5 confocal microscope (LEICA DMI 6000B) (Leica, Wetzlar, Germany).

References

1. Shih HJ, Chu KL, Wu MH, Wu PH, Chang WW, Chu JS, et al. The involvement of MCT-1 oncoprotein in inducing mitotic catastrophe and nuclear abnormalities. Cell Cycle. 2012; 11: 934-52.

2. Hsu HL, Choy CO, Kasiappan R, Shih HJ, Sawyer JR, Shu CL, et al. MCT-1 oncogene downregulates p53 and destabilizes genome structure in the response to DNA double-strand damage. DNA Repair (Amst). 2007; 6: 1319-32.
3. Shih HJ, Chen HH, Chen YA, Wu MH, Liou GG, Chang WW, et al. Targeting MCT-1 oncogene inhibits Shc pathway and xenograft tumorigenicity. Oncotarget. 2012; 3: 1401-15.
4. Weng YS, Tseng HY, Chen YA, Shen PC, Al Haq AT, Chen LM, et al. MCT-1/miR-34a/IL-6/IL-6R signaling axis promotes EMT progression, cancer stemness and M2 macrophage polarization in triple-negative breast cancer. Mol Cancer. 2019; 18: 42.
5. Cassetta L, Noy R, Swierczak A, Sugano G, Smith H, Wiechmann L, et al. Isolation of Mouse and Human Tumor-Associated Macrophages. Adv Exp Med Biol. 2016; 899: 211-29.
6. Sauer KA, Scholtes P, Karwot R, Finotto S. Isolation of CD4+ T cells from murine lungs: a method to analyze ongoing immune responses in the lung. Nat Protoc. 2006; 1: 2870-5.
7. Hori S. Lineage stability and phenotypic plasticity of Foxp3(+) regulatory T cells. Immunol Rev. 2014; 259: 159-72.
8. Chen HM, Tsai CH, Hung WC. Foretinib inhibits angiogenesis, lymphangiogenesis and tumor growth of pancreatic cancer in vivo by decreasing VEGFR-2/3 and TIE-2 signaling. Oncotarget. 2015; 6: 14940-52.
9. Crowe AR, Yue W. Semi-quantitative Determination of Protein Expression using Immunohistochemistry Staining and Analysis: An Integrated Protocol. Bio Protoc. 2019; 9.
